# Structural basis for antibody recognition of the proximal MUC16 ectodomain

**DOI:** 10.1186/s13048-024-01373-9

**Published:** 2024-02-19

**Authors:** Kwangkook Lee, Kay Perry, Mengyao Xu, Irva Veillard, Raj Kumar, Thapi Dharma Rao, Bo R Rueda, David R Spriggs, Oladapo O Yeku

**Affiliations:** 1 Division of Hematology & Oncology, Department of Medicine, Massachusetts General Hospital-Harvard Medical School, Boston, MA USA; 2 Department of Chemistry and Chemical Biology, Argonne National Laboratory, NE-CAT, Cornell University, Building 436E, 9700 S. Cass Avenue, Argonne, IL 60439 USA; 3 Memorial Sloan Kettering Cancer Center, New York, NY 10065 USA; 4 Department of Obstetrics and Gynecology, Vincent Center for Reproductive Biology, Massachusetts General Hospital, Boston, MA 02114 USA; 5 Division of Hematology and Oncology, Massachusetts General Hospital, Boston, MA USA

**Keywords:** MUC16, CA125, Ovarian cancer, Antibody drug conjugate, Bispecific T-cell engager, Chimeric antigen receptor-T cells, Crystal structure

## Abstract

**Background:**

Mucin 16 (MUC16) overexpression is linked with cancer progression, metastasis, and therapy resistance in high grade serous ovarian cancer and other malignancies. The cleavage of MUC16 forms independent bimodular fragments, the shed tandem repeat sequence which circulates as a protein bearing the ovarian cancer biomarker (CA125) and a proximal membrane-bound component which is critical in MUC16 oncogenic behavior. A humanized, high affinity antibody targeting the proximal ectodomain represents a potential therapeutic agent against MUC16 with lower antigenic potential and restricted human tissue expression.

**Results:**

Here, we demonstrate the potential therapeutic versatility of the humanized antibody as a monoclonal antibody, antibody drug conjugate, and chimeric antigen receptor. We report the crystal structures of 4H11-scFv, derived from an antibody specifically targeting the MUC16 C-terminal region, alone and in complex with a 26-amino acid MUC16 segment resolved at 2.36 Å and 2.47 Å resolution, respectively. The scFv forms a robust interaction with an epitope consisting of two consecutive β-turns and a β-hairpin stabilized by 2 hydrogen bonds. The V_H_-V_L_ interface within the 4H11-scFv is stabilized through an intricate network of 11 hydrogen bonds and a cation-π interaction.

**Conclusions:**

Together, our studies offer insight into antibody-MUC16 ectodomain interaction and advance our ability to design agents with potentially improved therapeutic properties over anti-CA125 moiety antibodies.

**Supplementary Information:**

The online version contains supplementary material available at 10.1186/s13048-024-01373-9.

## Background

The tethered mucin MUC16 protein is physiologically present and intricately controlled in reproductive, respiratory and corneal tissue to protect the epithelium by forming a biological mucosal barrier at the apical surface against hostile environmental conditions and pathogenic infections [1–4]. However, in several malignancies including ovarian, breast, lung, and pancreatic cancers, it has been reported that overexpression of MUC16 can promote unfavorable characteristics of cancer cells, including changes in cell-to-cell communication, enhanced proliferation, increased accumulation of cancer cells in the G2/M phase with apoptosis resistance, and tumor metastasis. MUC16 overexpression has also been shown to facilitate tumor immune escape via direct suppression of natural killer (NK) and macrophages [4–9].

MUC16 is composed of 3 major domains and the following subdomains; a heavily glycosylated extracellular region including an N-terminal portion, a tandem-repeated domain interspersed with sea urchin Sperm, Enterokinase, and Agrin (SEA) domain, and a carboxyl-terminal domain. The tandem repeat region encodes the CA125 antigen, a complex, O-glycosylation enhanced epitope that is the cognate receptor for the mesothelin protein [10–13]. The carboxyterminal sequence can be further divided into three subdomains composed of an extracellular 61 amino acid juxtamembrane (ectodomain) portion, a transmembrane (TM) region, a 31 amino acid cytoplasmic-tail domain harboring potential phosphorylation sites, and an ezrin binding domain [14–16].

The limited tissue expression of MUC16 has made it an attractive candidate for antibody-based, targeted therapy development in high grade serous ovarian cancer (HGSOC) [17–21]. However, much of this activity has utilized antibodies targeting the tandem repeat region. This strategy has two significant shortcomings: (a) the tandem repeat protein is present in the circulation, acting as an antigen sink and (b) the shed CA125 region promotes off-target effects particularly on mesothelin-expressing surfaces [18, 19]. Prior therapeutics like abagovomab, oregovomab, and DMUC (Genentech), have targeted the tandem repeat portion of MUC16 [14, 19, 22]. The OC125/M11 epitopes present in the tandem repeat region are dependent on folding and enhanced by glycosylation processes which have limited potential for antibody targeting of MUC16 [23]. Our prior work suggests that targeting non-CA125 protein epitopes in the proximal MUC16 ectodomain (MUC16^ecto^) using a murine antibody (m4H11) may still block MUC16 related oncogenic functions [24–26]. The sequences of the membrane-proximal MUC16 SEA domains are more divergent than those of distal SEA domains, and because mAb 4H11’s epitope is only partially conserved in other SEA domains it is assumed that soluble CA125 would not act as a sink. To mitigate the issues surrounding the use of murine antibodies as human drugs, we set out to engineer and characterize a humanized antibody version against MUC16^ecto^. In this report, we evaluated matrigel invasion, antibody drug conjugate (ADC) killing, and Chimeric Antigen Receptor (CAR)-T cell therapy. Finally, we carefully explored the structural interactions between the MUC16 ectodomain and a humanized version of 4H11 antibodies against MUC16^ecto^ targets. We describe the crystal structures of a single chain h4H11-scFv and describe its interaction with MUC16^ecto^ composed of 26 residues (^31th^LQNFTLDRSSVLVDGYSPNRNEPLTG^6th^; numbering from TM) to understand the binding mechanism through the comparison of the apo and the MUC16^ecto^-bound scFv at 2.36 Å and 2.47 Å resolutions, respectively. Our results reveal conformational differences in the complementarity-determining regions (CDRs) of the free and MUC16-bound 4H11 scFv. This information can be utilized to improve the therapeutic potential of antibody-based therapies.

## Results

### Humanization of 4H11 antibody

**Fig. 1:**
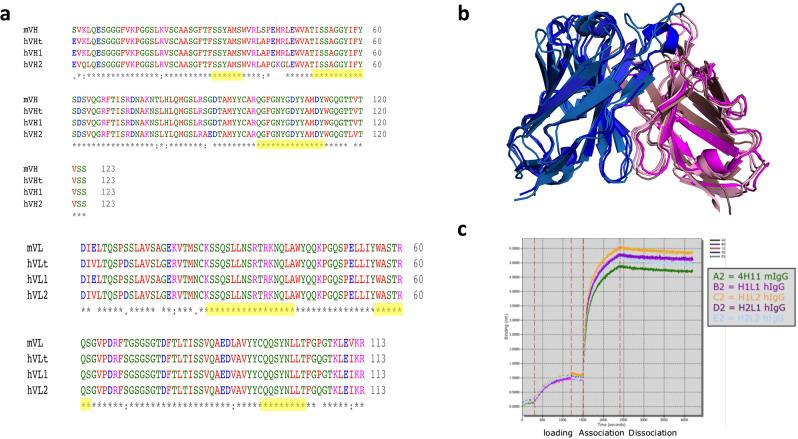
Humanization of 4H11. **(a)** Sequence alignment of; 4H11, human frame template and humanized h4H11 heavy and light chains, mV_H_ and mV_L_, heavy chain and light chain of 4H11 mouse IgG1, hV_H_t, the framework template of human heavy chain (4dtg), hV_L_t, the framework template of human light chain (3O2d). hV_H_1 and 2, hV_L_1 and 2, and the humanized heavy chain and light chain of 4H11. Yellow highlighted sequences are CDRs. Humanized amino acids indicated in red letters. **(b)** Structural overlay of 4H11 and humanized antibody structures. The structures of 4H11 and humanized antibody (4H11, hH1L1, hH1L2, hH2L1, hH2L2) were simulated by ABodyBuilder. 4H11 heavy (sky blue) and light (pink) chains. hH1L1 heavy (TV blue) and light (dirty violet) chains. hH1L2 heavy (marine) and light (violet purple) chains. hH2L1 heavy (deep blue) and light (purple) chains. hH2L2 heavy (density) and light (deep purple) chains. **(c)** Binding kinetics of 4H11 and humanized h4H11 antibodies by ForteBio Octet analysis

To form the basis for clinical development, the 4H11 murine antibody (m4H11) was first modified to provide a CAR for clinical application [20]. This m4H11 antibody had the best binding characteristics from our original antibody campaign and was used as the basis of the humanized development [24]. In collaboration with Eureka Therapeutics Inc (Emeryville, CA), the sequence of the m4H11 antibody was used to search international IMmunoGeneTics (IMGT) database for the closest human antibody framework, and antibody structure modelling was performed by ABodyBuilder. Two alternative humanized heavy chains (H1 and H2) and two light chains (L1 and L2) were designed in this manner (Fig. 1a). We hypothesized that the designed amino acid changes would not affect the binding affinity of the antibody. Using the same simulation model, the predicted structures of the humanized antibodies are very close to the structure of the original antibody m4H11 (Fig. 1b). The affinities of the resulting antibodies were assessed against MUC16 peptides from the MUC16^ecto^ to establish the avidity of the modified antibodies (Fig. 1c**)**. All 4 of the human heavy chain and light chain combinations demonstrated persistent avidity for the MUC16 peptide target. While avid binding appears similar, the monovalent binding affinities of the murine and humanized antibody may be different, and this was not evaluated.

### Functional analysis of the h4H11 antibody

**Fig. 2:**
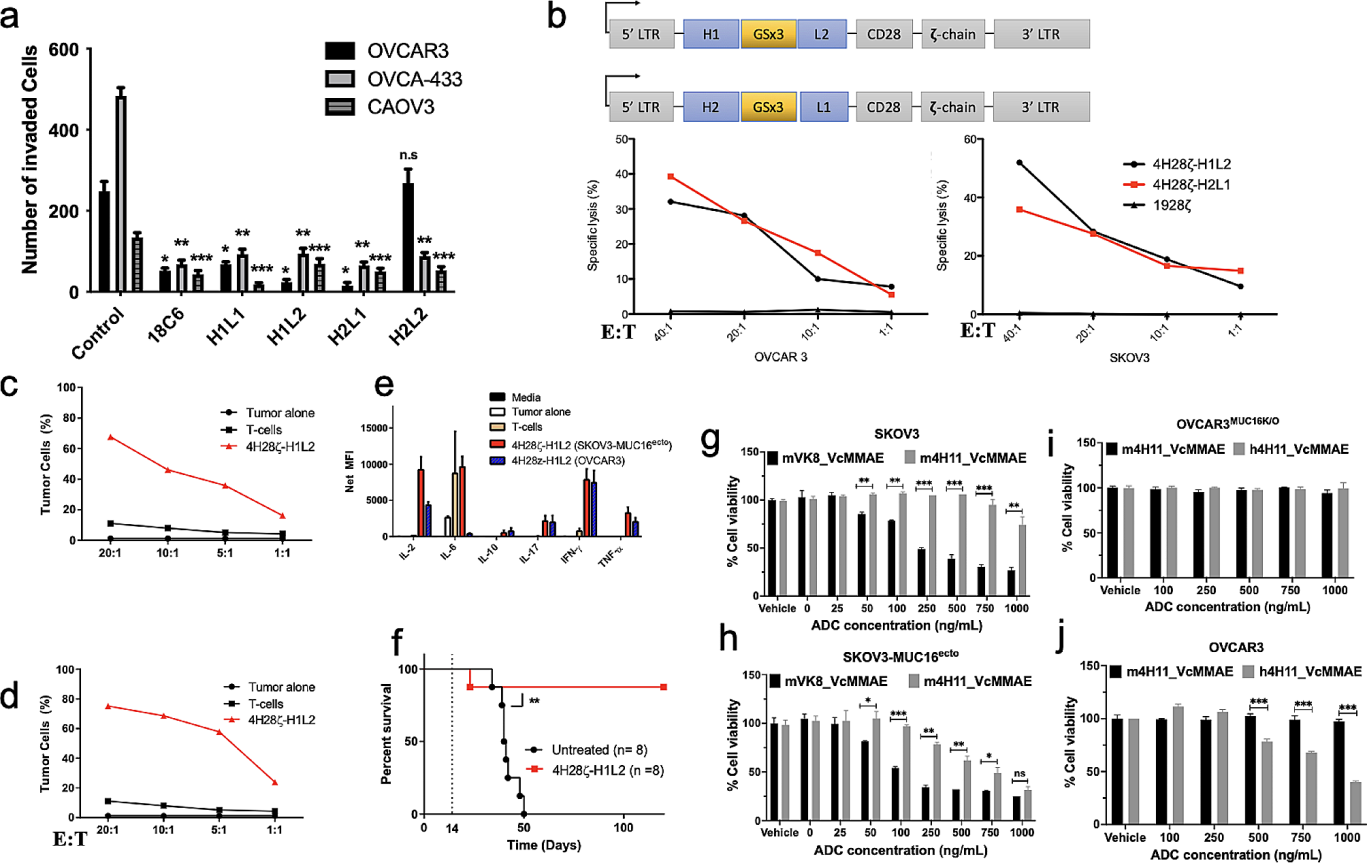
Functional characterization of humanized Muc16^ecto^ antibody. **(a)** Matrigel invasion assays performed with or without the addition of anti-MUC16^ecto^ antibodies. The antibody 18C6 was used as the positive control and decreased inhibition of all three cell lines. Humanized anti-MUC16^ecto^ 4H11 antibodies H1L1, H1L2, H2L1, and H2L2 were tested. All antibodies showed statistically significant inhibition of Matrigel invasion except OVCAR3 cells treated with H2L2. *p* < 0.005; * compared to OVCAR3 control, ** compared to OVCAR-433 control, *** compared to CAOV3 control, n.s: not significant. **(b)** 4 h Cr release cytotoxicity assays conducted with MUC16^ecto^ – directed second-generation CAR T-cells derived from H1L2 and H2L1 scFv sequences at the indicated effector to target (E:T) ratios. Both CAR T-cells showed dose-dependent cytotoxicity against OVCAR3 and SKOV3- MUC16^ecto^ tumor cells. **(c)** H1L2 4H11 CAR T-cells co-cultured with OVCAR3 cells and **(d)** SKOV3- MUC16^ecto^ tumor cells and evaluated for cytotoxicity after 72 h of coculture. **(e)** Cytokine analysis of H1L2 4H11 CAR T-cells co-cultured with SKOV3- MUC16^ecto^ or OVCAR3 tumor cells for 72 h. **(f)** 8–12-week-old female NSG mice were inoculated with SKOV3- MUC16^ecto^ tumor cells i.p. and treated with H1L2 4H11 CAR T-cells on day 14. Animals were subsequently monitored for development of ascites or signs of distress. ** *p* < 0.005. Animal experiments were performed with 4 animals per treatment group and performed twice (2 biologic replicates). **(g)** SKOV3, **(h)** SKOV3- MUC16^ecto^**(i)** OVCAR3^MUC16KO^, and **(j)** OVCAR3 tumor cells were treated with increasing concentrations of murine VK8, m4H11, or h4H11 MMAE-ADCs for 72 h. and evaluated for cytotoxicity. Data are plotted as means ± SEM from three independent measurements, ns, not significant, *p < 0.05, **p < 0.01, ***p < 0.001. Coculture experiments were performed with 2–3 technical replicates and 3 biological replicates unless otherwise stated

We previously illustrated that ability of the m4H11 anti-MUC16^ecto^ antibodies to recognize cellular MUC16 expression in Fluorescence Activated Cell Sorting (FACs), CAR-T cell application, and blocking of matrigel invasion [24, 25, 27, 28]. We next set out to evaluate the therapeutic potential of humanized 4H11 (h4H11) against MUC16^ecto^ tumor cells using a variety of therapeutic modalities. First, we examined the ability of H1L1, H1L2, H2L1, and H2L2 h4H11 antibodies to inhibit Matrigel invasion of MUC16-positive tumor cell lines; OVCAR3, OVCA-433, CAOV3. The indicated cell lines were incubated with 10 µg/ml of murine 18C6 antibody, H1L1, H1L2, H2L1, or H2L2 for 48 h. The number of invading cells in the absence (control) or presence of each of the antibodies are enumerated in Fig. 2a. Using the murine anti-CA125 antibody 18C6 as a positive control, we found that h4H11 H1L1, H1L2, and H2L1 antibodies significantly inhibited migration of OVCAR3, OVCA-433 and CAOV3 cells compared to untreated control tumor cells (*p* < 0.005; * compared to OVCAR3 control, ** compared to OVCAR-433 control, *** compared to CAOV3 control, n.s: not significant). Interestingly, the antibody h4H11 H2L2 inhibited invasion of OVCA-433 and CAOV3 cell lines (*p* < 0.0001 and *p* = 0.001) but not invasion by OVCAR3 cells. This difference could potentially be due to differences in antigen density between the different cell lines or other cell-line specific factors that could influence matrigel invasion. Since our aim was to select candidates with the most robust activity across multiple cell lines, we focused our subsequent round of evaluations on h4H11 H1L2 and H2L1. Next, we used the variable heavy and light chain sequences (scFv) of h4H11 H1L2 and H2L1 to generate second-generation CD28-costimulated CAR T-cells; 4H28ζ-H1L2 and 4H28ζ-H2L1 respectively (Fig. 2b). H1L2 and H2L1 were selected for CAR testing because both antibodies outperformed the other two in our matrigel inhibition assays. OVCAR3 and SKOV3-MUC16^ecto^ tumor cells were co-cultured with 4H28ζ-H1L2 or 4H28ζ-H2L1 for 4 h and assessed for cytotoxicity using a chromium (^51^Cr) release assay. Both 4H28ζ-H1L2 and 4H28ζ-H2L1 showed dose-dependent cytotoxicity against OVCAR3 and SKOV3-MUC16^ecto^ tumor cells over a range of effector to target ratios (E:T). We did not detect any significant cytotoxicity using control CD19-directed CAR T-cells (Fig. 2b). Due to the similarity in efficacy between 4H28ζ-H1L2 and 4H28ζ-H2L1, we used 4H28ζ-H1L2 for the remainder of our experiments. We evaluated cytotoxicity of 4H28ζ-H1L2 over 72 h against OVCAR3 (Fig. 2c) and SKOV3-MUC16^ecto^ cells (Fig. 2d) and found significant dose-dependent cytotoxicity compared to untransduced T-cells (*p* < 0.05). Cytokine analysis of 4H28ζ-H1L2 cocultured with SKOV3-MUC16^ecto^ and OVCAR3 over 72 h showed increased IL-2, IL-17, IFN-γ, and TNF-α secretion (Fig. 2e). To evaluate the in vivo efficacy of 4H28ζ-H1L2, we treated SKOV3-MUC16^ecto^ tumor-bearing female mice 14-days after they had been inoculated with tumor cells (intraperitoneal). As shown in Fig. 2f, **4H28**ζ-H1L2 significantly prolonged survival in treated mice. Finally, we evaluated MMAE conjugated H2L1 antibody-drug conjugates against SKOV3-MUC16^ecto^ and SKOV3 tumor cells. H2L1 ADCs mediated cytotoxicity in MUC16 positive SKOV3-MUC16^ecto^ cells but not MUC16 negative SKOV3 cells (Fig. 2g, h). Of note, we also examined a CA125 epitope binding antibody, VK8. Both the m4H11 ADC against the MUC16 ectodomain and VK8 ADC, directed at the CA125 antigen had direct killing effect against the SKOV3 cells transfected for MUC16 expression. In the control SKOV3 cells negative for both CA125 epitopes, VK8 ADCs unexpectedly showed increasingly dramatic off target killing as shown in Fig. 2g. Similarly, when we examined the effect of the human 4H11, we demonstrated that this antibody does not have significant off target activity in OVCAR3 cells engineered to remove MUC16 expression via CRISPR/CAS9 (Fig. 2i) while retaining efficacy against wild type OVCAR3 (Fig. 2j). The murine 4H11 ADC was used as an active control but not to serve as a direct comparator to the human 4H11 ADC.

### Construction and optimization of 4H11-scFv and MBP-MUC16^ecto^

To better understand the structure-function relationship of the humanized MUC16 ectodomain antibodies, we designed the H2L1 4H11-scFv consisting of the variable heavy (V_H_) and light chain (V_L_) of its parent humanized IgG connected by a repeated glycine-serine linker (Fig. S1). The linker length, composed of 25 amino acids (Gly-Gly-Gly-Gly-Ser)_5,_ showed high stability and correct orientation as a monomeric form with retained MUC16 binding. The scFv, [V_H_-linker (GGGGS)_5_-V_L_], was secreted into the culture media under the control of the signal peptide located at the N-terminal and then, purified as pure homogeneous monomers using Ni-affinity and SEC. The short MUC16^ecto^ domain, composed of 26 peptides was fused with a MBP to facilitate its stability and crystallization. The MBP fusion protein was recombinantly expressed in bacterial (*E. coli*) system and purified by a series of chromatographic procedures of MBP-affinity, Size Exclusion Chromatography (SEC) to high purity and prepared for the further experiments.

### The 4H11-scFv binds to MUC16^ecto^ and overexpressed-MUC16 on cancer cells

We first examined expression using two mammalian systems via lentiviral-based protein production by HEK293T/17, and transient protein production by ExpiCHO-S cells as a suspension culture. Both showed similar expression levels of up to ∼ 3 mg/Liter. Then, we purified proteins using Ni-affinity and SEC to examine the protein-protein interaction with MUC16^ecto^in vitro. It was important to confirm that the single chain construction had similar interactive properties to the full length h4H11 antibody. To confirm the 4H11-scFv and MUC16^ecto^ interactions, we performed four independent experiments, (1) analytical SEC, (2) in vitro pull-down assay, (3) isothermal titration calorimetry (ITC), and (4) visualization of Alexa-labeled scFv’s on the cell surface. We first conducted analytical SEC to characterize the interactions between 4H11-scFv and MUC16 (MBP-MUC16^ecto^) (Fig. S2). As expected, we could distinguish the scFv monomers and the scFv-MUC16^ecto^ complex, which was eluted 2.4 mL earlier than the excess scFv and 1.3 mL earlier than unbound MBP-MUC16^ecto^, respectively. Each eluted volume of the scFv, MBP- MUC16^ecto^, and the scFv-MBP MUC16^ecto^ complex were 12.6 mL, 11.5 mL, and 10.2 mL based on Superdex 75 10/300 GL, respectively (Fig. S2).

We next investigated our scFv binding to MUC16-overexpressing cancer cells through fixed or live cell imaging analysis and also evaluated if internalization took place. We prepared Alexa-fluorescence conjugated scFv (Alexa-4H11-scFv) to verify cell-surface binding of the scFv, while Alexa-350-WGA fluorescent dye was used to identify the cell membrane. Control cell lines, HEK293T/17 and SKOV3, showed no immunofluorescence on the fixed cell imaging (Fig. S1b). However, we found bright green fluorescent signal on the cell membranes of both OVCAR3 and SKBR3 which express MUC16, indicating that the scFv can specifically bind to native glycosylated MUC16 expressed on cancer cells (Fig. S1b). Live cell imaging was performed to track the internalization during multi-point time course (2 – 48 h). Confocal imaging revealed that the scFv could be localized in the cytoplasm, with gradual increase of enhanced fluorescence (Fig. S1c). We have previously reported that radiolabeled parent murine monoclonal antibody IgG-4H11 was readily internalized in OVCAR3 cells through the measurement of radioactively labeled antibody [24]. Therefore, the internalization of smaller 4H11-scFv is consistent with our earlier result. We performed ITC experiments as a label-free interaction analysis. We examined two types of MCU16^ecto^, one was a commercially synthesized 26 amino acids of MUC16^ecto^ and the other was recombinantly expressed MBP-MUC16^ecto^, to examine if the peptide itself functions as an antigen. The 4H11-scFv binds MBP-MUC16^ecto^ or the synthesized peptide with dissociation constant (*K*_*d*_) of ∼ 2 ± 1 nM or ∼ 1.4 ± 0.5 nM at pH 7.4, respectively (Fig. S1d, e). The complex form was further supported by an in vitro pull-down assay using 411-scFv as a prey and MBP-MUC16^ecto^ as a bait (Fig. S1f). Taken together, these results indicate that 4H11-scFv binds to MUC16-expressing cells, and suggests that the complex can be internalized, perhaps through MUC16-mediated endocytosis pathway [4].

### X-ray structure of 4H11 scFv in complex with MUC16^ecto^

**Fig. 3:**
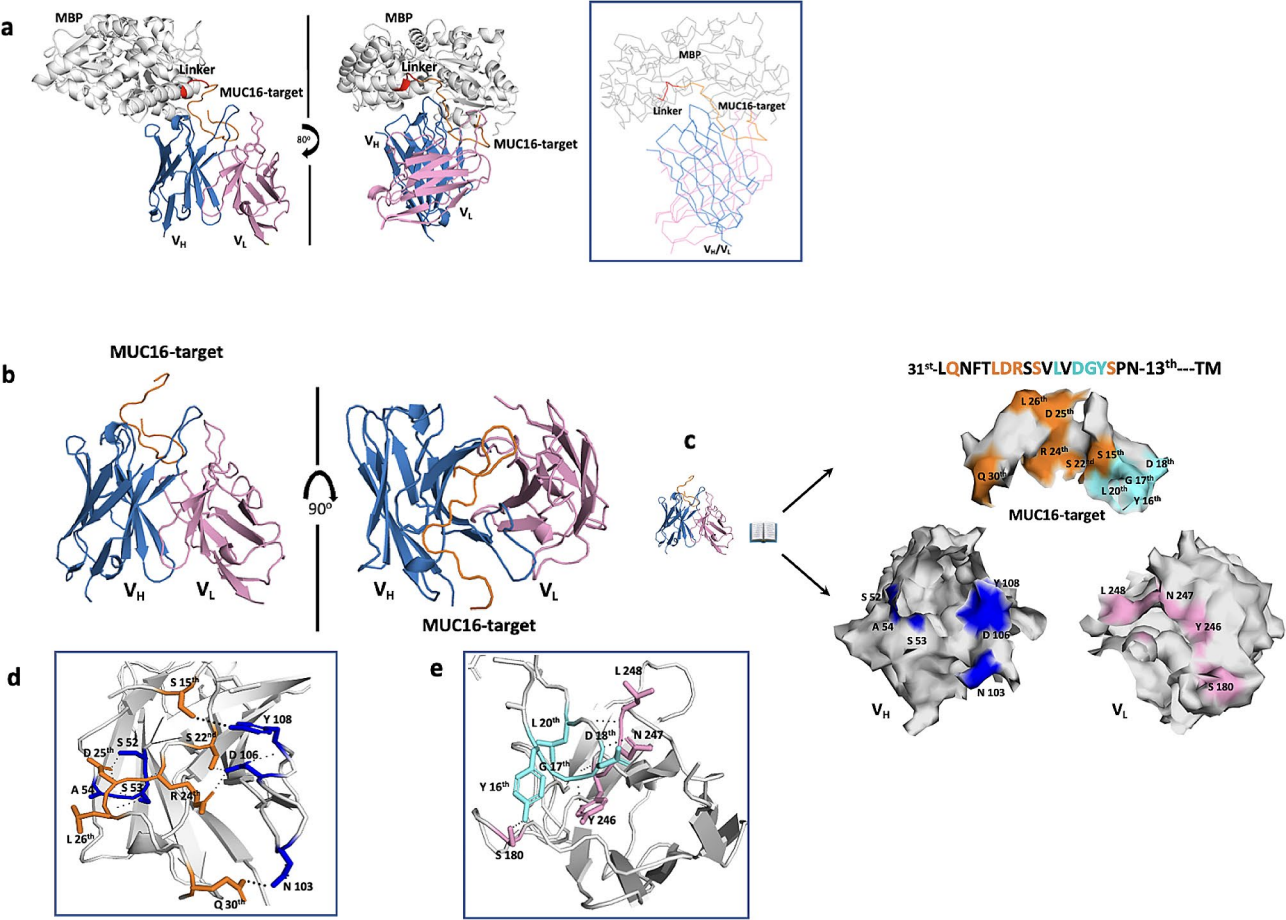
Structure of the scFv and MUC16 complex. (**a**) Cartoon representation of 4H11-scFv in complex with MBP-fused MUC16-target. The MBP was used to facilitate crystallization by stabilizing the MUC16 target peptide. MBP, maltose binding domain (grey); linker composed of NSSS (red dots); MUC16- target composed of 26 residues (orange); Heavy chain of 4H11-scFv (skyblue); linker composed of (GGGGS)5 repeats (black dots); Light chain of 4H11-scFv (pink). Line representation for clarity. (**b**) the interaction between V_H_, V_L_ and MUC16^ecto^ is enlarged for clarity. (**c**) An open book view of the interface residues between 4H11-scFv and MUC16-target highlighted in the box in panel e; The residues of MUC16 (S 15th, S 22nd, R 24th, D 25th, L 26th, and Q 30th) in orange color form the hydrogen bonds or salt bridges with V_H_ residues (S 52, S 53, A 54, N 103, D 106, and Y 108) in blue color, while the residues (Y 16th, G 17th, D 18th, and L 20th) of MUC16 in cyan color form the hydrogen bonds with V_L_ residues (S 180, Y 246, N 247 and L 248) in pink color. Amino acid numbering starts from the transmembrane (TM) region. (**d-e**) Interacting residues are labeled in close-up views of the interfaces. See Table S4, Fig S2 and S5 for versions of the detailed interactions

To further understand the molecular basis of the antibody binding interaction, we determined the crystal structures of both the 4H11-scFv and the 4H11-scFv-MUC16^ecto^ complex at 2.36 Å and 2.47 Å, respectively (Fig. 3a, Table. S1). The complexes were crystallized at pH 5.0, which is a physiologically relevant pH inside an endosome or in an hypoxic tumor environment. A complete structure of the complex was built except for two regions; the linker domain composed of Gly-Ser repeats and the C-terminal 7 residues (RNEPLTG) of MUC16^ecto^ sequences, that have no visible electron density probably due to high structural flexibility. Regardless, the 7 residues placed outside of the interface of the complex did not affect the interaction based on the structural analysis.

We found that MUC16^ecto^ directly interacts with CDR2-CDR3 on V_H_ through a relatively large interface area (∼ 617 Å^2^), covering residues in two β- turns (^31st^LQNFTLDRSS^22nd^) including the N29th glycosylation site (Fig. 3b, c). In contrast, the binding interface area for V_L_ was smaller (∼ 301 Å^2^), mainly including residues in the C-terminal loop including a surface β-hairpin (Fig. 3d). These structural findings at the interface region suggests that the V_H_ domain plays a dominant role in the interaction with MUC16. These interactions are enlarged for clarity to better illustrate the interaction residues **(**Fig. 3e**).**

### V_H_:V_L_ architecture in the free and MUC16-bound 4H11-scFv

**Fig. 4:**
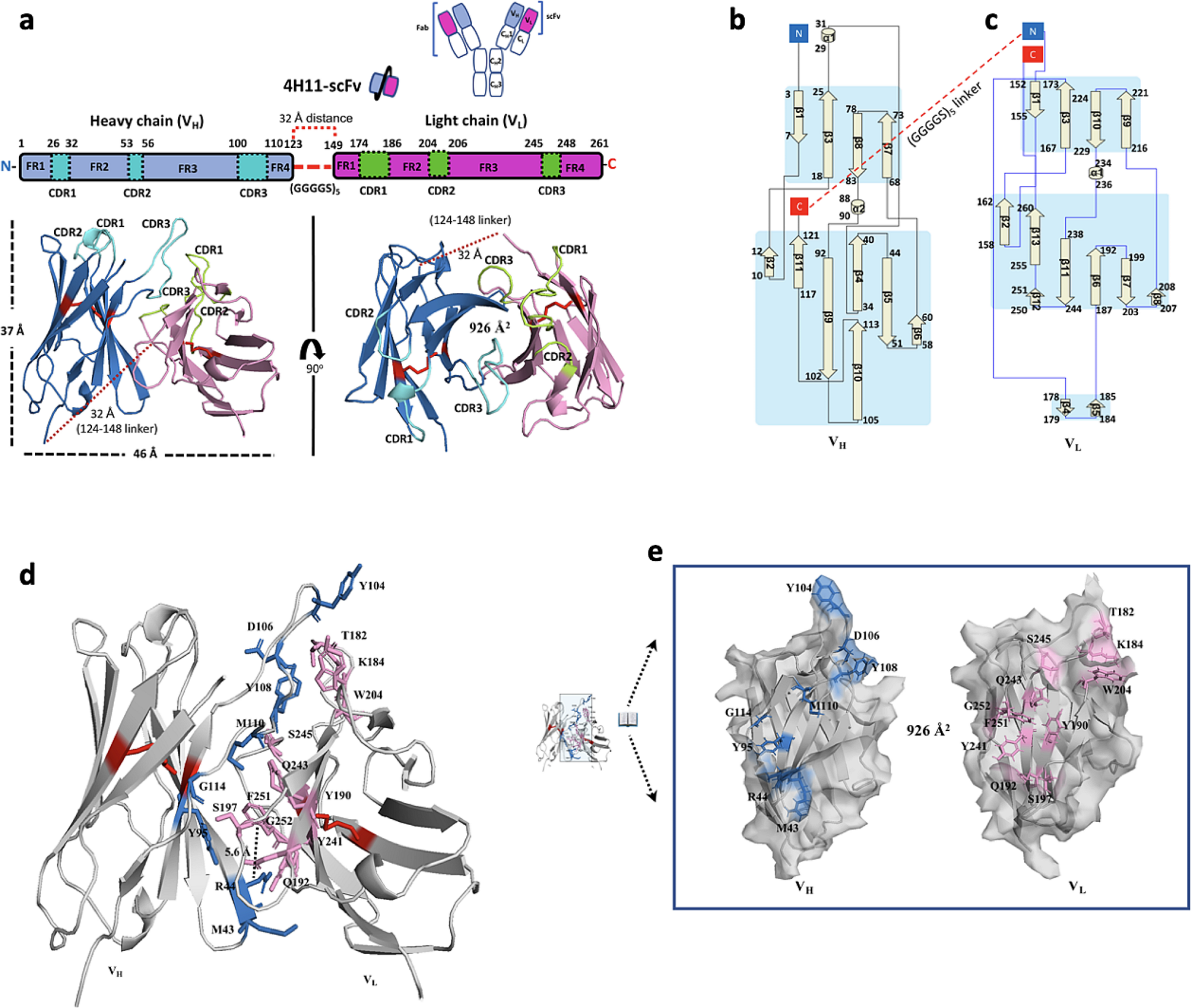
Overall structure of 4H11-scFv and interaction between V_H_ and V_L_. (**a**) Schematic diagram showing the domain structures of Heavy chain (Hc: 1-123) and Light chain (Lc: 149–261), as well as the positions of 3 CDRs (residue positions: 26–32, 53–56, 100–110) at V_H_ (cyan) and 3 CDRs (residue positions: 174–186, 204–206, 245–248) at V_L_ (forest) interacting with MUC16^ecto^. CDR: complementary determining region. The shortest distance from C-terminal of V_H_ to N-terminal of V_L_, the flexible linker (GGGGS)_5_ lengths (red dots) with no electron density map (residues 124–148), was 32 Å which corresponds to at least 13 amino acid-lengths. Each domain has one S-S bridge (red) (C22 – C96 at V_H_ and C171 – C242 at V_L_) for stable folding. The solvent-accessible area of the interface between V_H_-V_L_ was 926 Å^2^. (**b, c**) Secondary structure topology diagram of V_H_ (left panel) or V_L_ (right panel) of 4H11-scFv, the eleven sheets and two short helices for V_H_ and the thirteen sheets and one helix for V_L_ are represented. C-terminal (red) of V_H_ is connected to N-terminal (blue) of V_L_ via (GGGGS)_5_ linker (red dots). (**d**) Interacting residues at interface regions between V_H_ and V_L_ are colored as blue (V_H_ residues) and pink (V_L_ residues). The cationic sidechain of R44 (V_H_) form a favorable cation- π pair (black dot; 5.6 Å distance) with an aromatic sidechain of F251 (V_L_) to improve overall stability. (**e**) An open-book view of the interface with interacting residues. See Table S2 for detailed interacted residues

Each domain (V_H_ or V_L_) contains three CDRs and four framework regions (FRs) that support each CDR by strengthening antigen surface recognition. Structural analysis demonstrated that all six CDR regions are composed of 5 flexible loop-like coils, a short-helix (α_1_), and canonical disulfide bonds at the C22-C96 of V_H_ and C23-C94 of V_L_ that are formed to improve the correct folding and thermo-stability. The overall architecture of the scFv is ∼ 37 Å tall and ∼ 46 Å wide between the tips of neighboring loops (Fig. 4a). The V_H_-V_L_ complex are likely relatively independent of each other, and associate only through the interface. The V_H_ domain of the scFv is composed of 11 β strands (β1–β11) and 2 short helices (α_1−_α_2_), while 13 β strands (β1–β13) and one short helix (α_1_) for V_L_. The C-terminal of V_H_ is connected to the repeated Gly-Ser linker loop and following V_L_ begins from D149 residue (Fig. 4b, c). Our crystal structure also revealed that the straight distance from the C-terminal of V_H_ to N-terminal of V_L_ was ∼ 32.0 Å, corresponding to a distance of approximately 13 amino acids (∼ 2.7 Å per aa) (Fig. 4a **- c**). It is worth noting that the number of amino acids constituting the linker region between V_H_-V_L_ must be more than 13 residues to prevent an inactive or aggregated form due to the insecure space for interaction between V_H_-V_L_. A buried solvent-accessible area of 926 Å^2^ (calculated by PDBePISA v1.52) capable of forming hydrogen bonds, an energetically cation- π interaction (∼ 5.6 Å distance) between cationic sidechain (R44 at V_H_), and an aromatic sidechain (F251 at V_L_) may improve overall stability between V_H_-V_L_ (Fig. 4d, e).

We observed a dramatic conformational differences including reduced interactions and a new hydrogen bond between Q115 of V_H_ and S197 of V_L_ at the interface area between V_H_-V_L_ during ligand MUC16^ecto^ binding (Fig. S4f). This clearly shows the structural dynamics of 4H11-scFv with an interface flexibility depending upon ligand MUC16^ecto^ binding. The observed changes of CDR residues participating in the interaction with MUC16 seems to happen almost simultaneously with the changes of the interface between V_H_-V_L_ (Fig. S4). According to the reported molecular-dynamics-simulation study of antibodies, the timescale of CDR loop dynamics occurs on the micro- (10^− 6^) to millisecond (10^− 3^), while V_H_-V_L_ movements showing nanosecond (10^− 9^) timescale [29, 30]. This suggests the ligand binding of the scFv, although response speed of CDRs against antigen is slower than V_H_-V_L_ response, may offset its slower action and facilitate the conformational difference of V_H_-V_L_ interface for achieving the stronger affinity against MUC16^ecto^ antigen (explored below). We then wondered how the antigen may affect the thermo-stability of antibody. Using a fluorescence-based thermal shift assay, we found that the melting temperature (*T*_*m*_) of 4H11-scFv was increased by 12.5% (acidic pH) and 4.2% (neutral pH) in the presence of MUC16^ecto^ peptide, whereas MUC16^ecto^ peptide itself had no effect (Fig. S3). Interestingly, we found that independent melting points of each domain (V_H_ or V_L_) between pH 5.4 and pH 9.4, implying there is increased thermo-stability by ∼ 13% at acidic and ∼ 23% at neutral pH (Fig. S3c). In contrast, the scFv in complex with ligand MUC16^ecto^ showed only one melting point at each pH value. These findings imply that the apo scFv (V_H_-V_L_) has a higher independence at even pH 4.4 and unfolding of the scFv can be inhibited depending on the MUC16^ecto^ ligand.

### Dynamic rearrangement of the 4H11 scFv CDR’s during binding with the MUC16 ectodomain

**Fig. 5:**
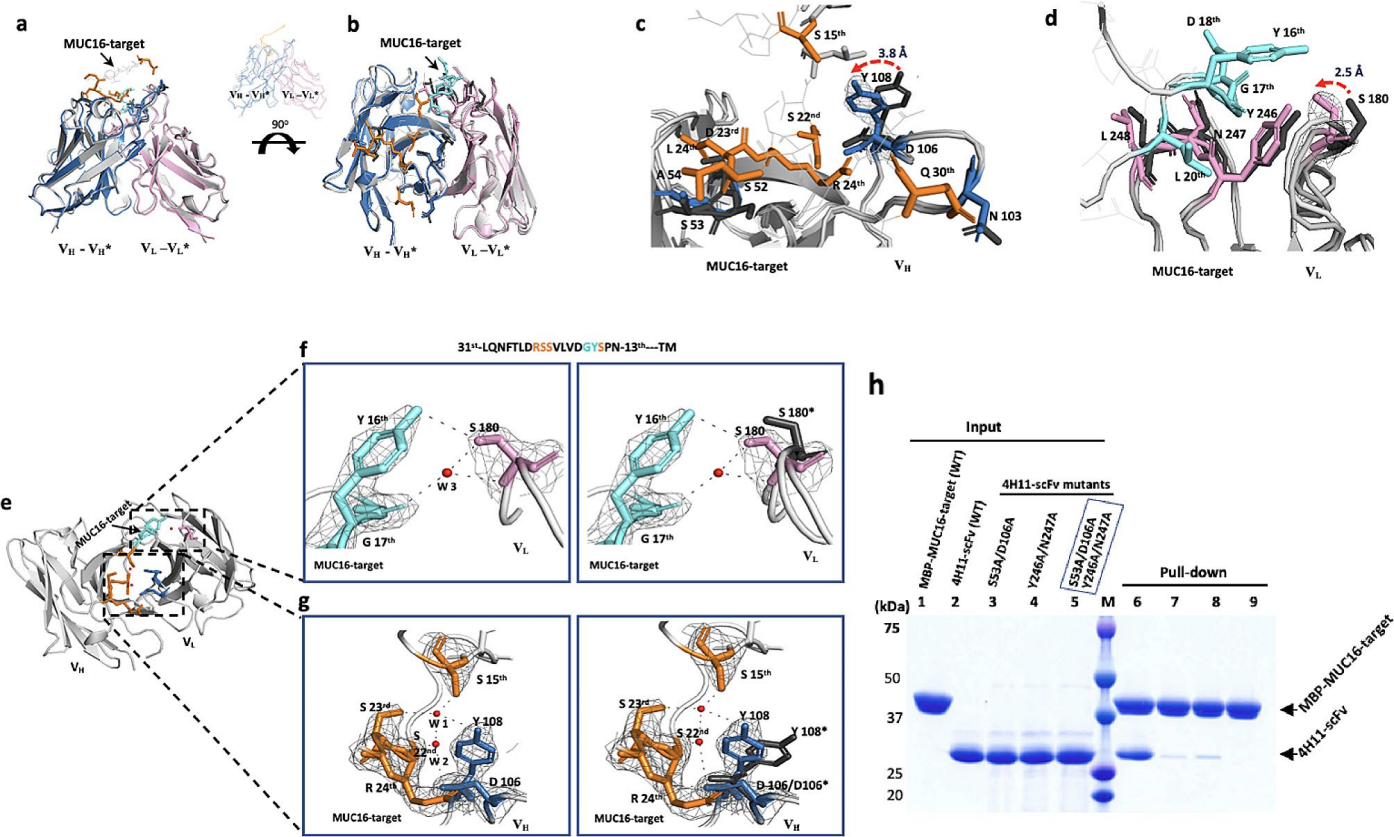
The binding modes of the 4H11-scFv rearrange the CDRs of the scFv. **(a, b)** The overall superpositions of the structures of the unbound and MUC16-target bound 4H11-scFv. The unbound V_H_*-V_L_* (grey), bound V_H_ (skyblue)-V_L_ (pink), MUC16-target (orange). The view directions in 5a and b are similar to those shown in Figs. 3a and 90° rotation of the complex about a horizontal axis (5b). (**c, d**) The close-up views of the interfaces between V_H_-V_H_* (c) or V_L_-V_L_* with the movement indicated by the red dotted arrow. The aromatic ring of Y108 with 2Fo-Fc map was moved to the left up to ∼ 3.8 Å and The OG of S 180 with 2Fo-Fc map was also moved to the left up to ∼ 2.5 Å. (**e-g**) The close-up views of the hydrogen bonds formed by water molecules (W1, W2, or W3) during the complex. Y16th and G 17th (cyan) of MUC16-target (f) and S 15th, S 23rd and R 24rd (orange) of MUC16-target (g) formed the multi-hydrogen bonds with V_L_ S180 and V_H_ Y108/D106. See Table S4 for detailed interactions. (**h**) Pull-down assay was performed using 4H11-scFv and mutants as a prey and the MBP-tagged MUC16 (26 residues) as a bait. After binding, the MBP resins were washed three times, and the bound proteins were released and subjected to SDS-PAGE. Lane 1–5: input proteins of MBP- tagged MUC16-target (lane 1), 4H11-scFv (lane 2), and the scFv mutants (lane 3–5); lane 6, pull- down as a control; lane 7–8, 4H11-scFv containing V_H_ double mutations (S53A/D106A) or V_L_ double mutations (Y246A/N247A); lane 9, the two double mutations (S53A/D106A, Y246A/N247A) of the scFv. The residues of MUC16 (S 15th, S 22nd, R 24th, D 25th, L 26th, and Q 30th) in orange color form the hydrogen bonds or salt bridges with V_H_ residues (S 52, S 53, A 54, N 103, D 106, and Y 108) in blue color, while the residues (Y 16th, G 17th, D 18th, and L 20th) of MUC16 in cyan color form the hydrogen bonds with V_L_ residues (S 180, Y 246, N 247 and L 248) in pink color. **(g-h)** Interacting residues are labeled in close-up views of the interfaces. See Table S2, Fig S3 and S4 for versions of the detailed interactions

Based on our structural model of the 4H11 scFv alone, we noted that specific sites in the 4H11 scFv underwent alteration when bound to MUC16^ecto^. After careful structural analysis of the scFv apo and bound forms, antigen binding-induced changes were identified (Fig. S4). We found structural changes in the CDR loop regions of the V_H_ and V_L_, corresponding with allosteric movements without overall distortion. Root-mean-square-deviation (R.M.S.D) between the backbone atoms of heavy chains (V_H_*: V_H_) or light chains (V_L_*: V_L_) with these changes were 0.454 or 0.355, respectively (Fig. S4c, Table. S2). Figure 5a and b show superimposed ribbon structures which highlight the changes in the heavy chain and light chain when bound to the MUC16 target sequence in two views. These movements are illustrated in Fig. 5c and d. In particular, the sidechain of V_L_ S180 was moved ∼ 2.5 Å, forming hydrogen bonds with Y16th and G17th of MUC16 (Fig. 5e **– f**, pink highlight). The aromatic ring of the V_H_ Y108 was also moved ∼ 3.8 Å after MUC16 binding, toward S15th and S23rd residues of MUC16 and Fig. 5e **- g**). We found these movements of Y108 and S180 are linked to water molecules (w1-w3) as illustrated in the (Fig. 5f **- g**). Three water molecules (w1-w3) contribute 5 pairs of hydrogen bonds through bridging D106 and Y108, and S180 with the MUC16 ectodomain (Fig. 5f **- g**). No water molecules at the specific positions were observed in the 4H11-scFv structure itself, probably because of the roles of water-binding residues at the V_H_-V_L_ interface, implying the water molecules may contribute to gain increased interactions.

Interestingly, preliminary CDR mutagenesis studies demonstrate that the antibody’s binding affinity is dramatically improved when V_H_ and V_L_ together, the V_H_-V_L_ complex, interacted with each other against antigen MUC16^ecto^ (Fig. 5h). The V_H_-V_L_ heterodimer showed much stronger affinity than its variants designed for blocking each binding capacity, although each could independently occupy the specific positions of MUC16^ecto^ as shown in the pull down (Fig. 5h). MUC16^ecto^ variants designed for blocking the interactions with V_H_ or V_L_ showed that V_H_ binding affinity may be more critical for interaction than V_L_ affinity to the antigen.

### The structure of MUC16^ecto^ is unique in humans, independent of glycosylation and conserved across phylogeny

**Fig. 6:**
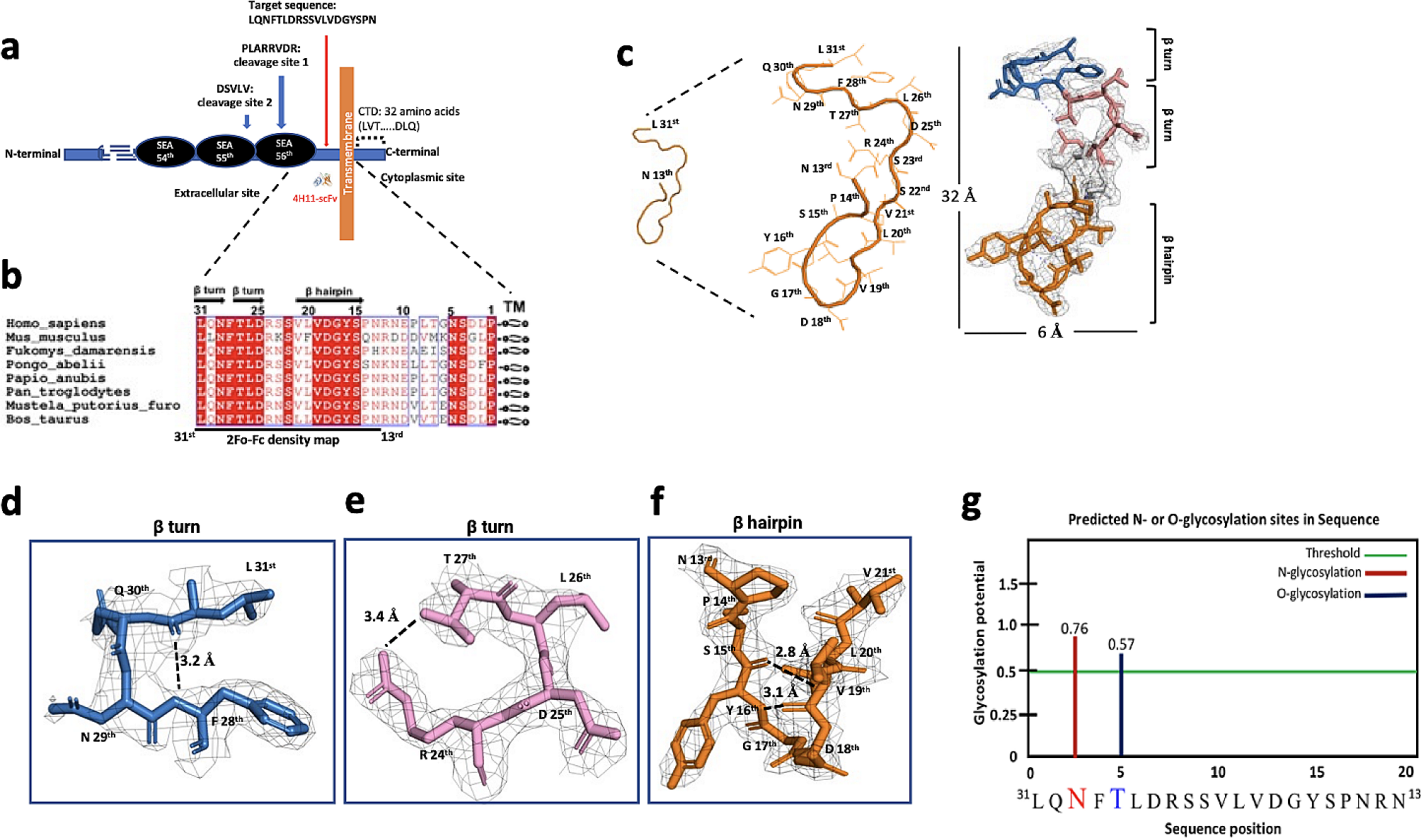
The target domain of 4H11-scFv is located at the juxta-membrane upward transmembrane (TM) of MUC16. **(a)** Schematic representation of MUC16 structure: MUC16 can be divided by three parts: N-terminal domain (∼ 22,000 amino acid in length), tandem repeat domains interspersed with Sea urchin sperm protein Enterokinase and Agrin (SEA) domain including potential cleavage sites (DSVLV and PLARRVDR) and C-terminal domain that is further divided into an extracellular juxtamembrane portion, a single–span TM and a cytoplasmic tail of 32 amino acid length. The 4H11-scFv targets to the juxta-membrane as shown target sequence (red arrow). **(b)** Amino acid sequence alignment of the juxta-ectodomain among 8 different species. Secondary structure of human MUC16 is shown on the top as double β turns-linker-β-hairpin structures. Sequence alignments were made using Clustal Omega and ESPript 3.0. **(c)** Crystal structure and representative electron density maps of the MUC16^ecto^ residues (L31st -N13th) that are complexed with 4H11-scFv. The stick representation with a 2Fo-Fc electron density map for MUC16^ecto^ contoured at 1.0 σ shows two β- turns and one β-hairpin structures. Two β-turns **(d, e)** and a β-hairpin **(f)** structures are highlighted in the box with distances of hydrogen bonds between F28th (-N) – L31st (-O), R24th (-NH2) –T27th (-OG1), V19th (-N) – Y16th (-O) and V19th (-O) – Y16th (-N). See Table S3 for version of the detailed Phi (φ), Psi (ψ) and Omega (ω). **(g)** The predicted glycosylation sites in 19 residues using web-server (http://crdd.osdd.net), N-or O-glycosylation sites; ”N” (red) or “T” (blue), respectively

Following proteolytic cleavage of MUC16 in vivo, MUC16 fragments have two independent biological elements: the shed “tandem repeat element” and “proximal retained component”, including the ectodomain, transmembrane domain and cytosolic sequences. We have established that the juxtamembrane adjacent to TM is targeted by h4H11, a region more proximal to the membrane, with potential therapeutic advantages **(**Fig. 6a**)**. To evaluate phylogenetic stability of this region, we performed the sequence alignment of the specific 31 amino acids (from L31st to P1st) among 8 different species to examine sequence conservation (Fig. 6b). The ectodomain was highly conserved with ∼ 94% identity and in particular, showed 100% identity on the 16 residues (from L31st to S15th) except for Mus musculus. A structural view of MUC16^ecto^ revealed that it is composed of two consecutive β-turns of ^31st^L∼F^28th^ and ^27th^T∼R^24th^ residues and a β-hairpin of 9 residues (Fig. 6c-f). As known, β-turns are one of the most common structural motifs in proteins and change the direction of the peptide backbone by nearly 180°, allowing the peptide chain to fold back into itself. The hydrophilic N29th and D25th might have a high propensity for the formation of β-turns due to their placement on solvent-exposed surfaces (Fig. 6d and e). In addition to their role in protein folding, it is worth noting they can also serve as recognition motifs for protein-protein interactions (PPI), because the MUC16 C-terminal portion may function as a transcriptional motif in the nucleus [26, 31, 32]. We further analyzed the torsional angles (*Φ*_*i* + 1_, *ψ*_*i* + 1_, *Φ*_*i* + 2_, and *ψ*_*i* + 2_) in residues *I* + 1 and *i* + 2 and additional omega (ω) from L31st to P14th except for phi (φ) and psi (ψ) of the L31st owing to no calculation (Table. S3). The first β-turn seems to be type-I and the second β-turn as a mirror image of the backbone conformation of type-I based on the torsional angles. The β-hairpin is a simple motif that consists of two β-strands, oriented in an antiparallel direction (the N-terminus of one sheet is adjacent to the C-terminus of the next). It could be stabilized by two inter-hydrogen bonds between V19th (-N) – Y16th (-O) at 2.8 Å distance and V19th (-O) – Y16th (-N) at 3.1 Å distance, respectively (Fig. 6f). We also considered whether glycans in MUC16^ecto^ could be involved in antibody binding areas. As MUC16 is heavily glycosylated, we predicted N- or O-glycosylation sites based on the MUC16^ecto^ sequence using webserver (http://crdd.osdd.net) and confirmed our prior observation that N29th and T27th are likely to be glycosylated in vivo **(**Fig. 6g**)**. Furthermore, the N29th amino acid was involved in N-glycosylation motif known sequence “N-X-S/T” [33]. These potential glycosylation residues support interaction with the scFv in vivo, however these two residues showed no direct interactions in our structures. Taken together, the structural motifs of MUC16 ectodomain may play important roles for PPI in the cytosol or nucleus.

## Discussion

The tethered mucins are important biological molecules that are overexpressed in many human cancers. Mucins like MUC1, MUC4 and MUC16 have been suggested as potential targets for diagnosis and treatment. Each of the tethered mucins appear to transform fibroblasts under appropriate conditions [34]. The functions of mucins appear to be related to extensive glycosylation with attendant regulation of growth receptors, and cell surface behaviors through protein interactions within clathrin-coated pits [25]. Antibody-based strategies against mucin derived tumor associated antigens have been proposed for therapeutics, though none have successfully completed development [35]. Clinical MUC16 targeting with antibodies against the tandem repeat which is found in circulation have been explored [19]. A more proximal target in the noncirculating ectodomain from tethered mucins may have better pharmacologic properties for therapeutics development.

We have shown that MUC16^ecto^ overexpression is related to oncogenic behavior with increased invasion and metastatic potential [4, 6, 9, 36]. The multiple tandem repeat region of tethered mucins like MUC16 are highly immunogenic and most anti-MUC16 antibodies have targeted the shed form of MUC16 (known as CA125). Therapeutics like abagovomab, oregovomab, and DMUC (Genentech) targeted the shed form of MUC16 [14, 19, 22]. Antibodies like h4H11 that avidly bind the MUC16 juxtamembrane domain may represent a potentially superior therapeutic with high stability and specificity. The comparatively greater off target effects of the M11 family member VK8 suggest that the tandem repeat target for MUC16 might be a greater problem in clinical development, although the mechanism for this remains to be elucidated. The overexpressed MUC16 protein is generally found anchored on cell surface in clathrin lined pits and undergoes cleavage over time into two independent fragments: the “retained ectodomain” including the transmembrane-cytoplasmic sequences and “shed tandem repeat form” bearing multiple 156 amino acid tandem repeats. The tandem repeat fragment is found in the circulation where it is detected by the OC125 antibody. The characteristic biology of the unshed form is less understood. Although intact MUC16 seems to be the dominant form on high grade serous ovarian cancer, our previous data suggest that a small fraction of cell surface MUC16 molecules may lack tandem repeat immunoreactivity but retain reactivity to MUC16^ecto^ targeted antibodies like 4H11 [24]. We have also shown that the murine 4H11 antibody against MUC16^ecto^ demonstrates favorable internalization properties compared to the CA125 antigen [24]. The human 4H11 (h4H11) was designed as a humanized homolog antibody with high binding affinity. We propose that the h4H11scFv-MUC16^ecto^ complex, like the full antibody, is probably translocated during trafficking into the cell by endocytosis [34]. Cell surface MUC16 may re-enter into the cell through cleavage dependent endocytosis which might explain the efficiency of ectodomain targeting compared to tandem repeat targeting [34, 37]. For these studies, we created the h4H11-scFv with similar binding properties to the parent antibody. The scFv is stable at acidic environments and may promote better in vivo tumor penetration and rapid serum clearance due to the roughly 5 times smaller size (∼ 28 kDa) compared to the parent antibody (∼ 150 kDa). We used a MUC16^ecto^ target sequence that was initially designed with 26 amino acids, however only 19 residues (^31st^L∼N^13rd^) were identified by crystal structure, probably owing to high flexibility of 7 residues shown as non-bound portion (which have been implicated by others as a potential nonenzymatic cleavage site), but our results do not address this issue [31].

We delved into the conformational dynamics occurring at the interface between the V_H_ and V_L_ domains of the 4H11-scFv antibody upon interaction with the MUC16 antigen. This analysis sheds light on the structural changes that underlie the antibody-antigen recognition process, however, caution needs to be applied when extrapolating affinity data generated using peptide and MBP fusion as this may not fully recapitulate binding to the native MUC16 in situ. Initially, we observed that the 4H11-scFv antibody, in its unbound state, exhibited robust stability attributed to the extensive interaction between the V_H_ and V_L_ domains. Our X-ray structure revealed the presence of 11 hydrogen bonds at this interface, reinforcing the stability of the unbound antibody (Fig. 3 and Table. S4). However, upon binding to the MUC16 antigen, we observed conformational differences at the V_H_-V_L_ interface (Table. S2). These changes were characterized by the disruption of 6 hydrogen bonds, each involving altered atom-to-atom distances. Significantly, a new hydrogen bond emerged between Q196 (red) of V_H_ and S197 of V_L_, underscoring the dynamic nature of the interface during MUC16 binding. To visually represent these changes, we employed a superimposed line model (Fig. S4a-b) that showcased the structural disparities between the bound and unbound states. The root-mean-square-deviation (R.M.S.D) values for the superimposed V_H_ and V_L_ domains were 0.454 and 0.355, respectively, highlighting the substantial alterations driven by antigen binding (Fig. S4). Further exploration of specific residues provided deeper insights into the conformational dynamics. For instance, the hydrogen bond between Y104 (V_H_) and K184 (V_L_) was disrupted due to the movement of K184 by 2.2 Å. This shift was accompanied by an orientation change in the aromatic ring of Y104, resulting in a measured distance of 4.4 Å between the relocated K184 and Y104, with no direct contact (Fig. S4). Y108 (V_H_) underwent a significant 3.3 Å shift towards MUC16, leading to the disruption of previous hydrogen bonds with V_L_ residues (T182, Q243, and S245). Instead, new hydrogen bonds were formed via water-mediated interactions, further emphasizing the adaptability of the interface. Notably, a novel hydrogen bond was established between Q115 (V_H_) and S197 (V_L_) upon MUC16 binding (Fig. S4f). This interaction necessitated a ∼ 180° rotation of the sidechain of Q115, facilitating the formation of the hydrogen bond with S197. It is important to note that our observed conformational difference may be an artefactual result of the scFv architecture and different results may be obtained if a Fab or IgG antibody is used. In summary, our study provides a comprehensive understanding of the conformational dynamics at the V_H_-V_L_ interface during MUC16 binding. These intricate changes underscore the adaptability of the antibody structure in response to antigen interaction, with potential implications for the design of antibodies with enhanced specificity and stability for therapeutic applications.

## Conclusions

The retained portion of MUC16/CA125 (MUC16^ecto^) represents a viable therapeutic target for high grade serous ovarian cancer and other solid tumor malignancies. Antibodies recognizing this target with high avidity are substrate for generation of ADC, BiTE and CAR T cells. We have described a novel antibody derived 4H11-scFv apo structure, its antigen MUC16^ecto^-bound complex structure, and antibody dynamics depending upon antigen MUC16^ecto^. To our knowledge, this structure of MUC16^ecto^, composed of 19 residues is released as the first human MUC16 structure and the first retained ectodomain for any tethered mucin. Most antibodies can reorganize their CDRs to engage antigens [38]. Indeed, binding to the antigen MUC16^ecto^ appears to trigger a series of rapid conformational rearrangements of both the CDRs and the structural movements of V_H_-V_L_ interface. Taken together, our study provides a platform and strategy for antibody optimization studies targeting the MUC16 ectodomain and a structural mechanism of 4H11-scFv – MUC16 interaction. These studies are an essential step in MUC16 immunotherapeutic agent development for clinical trials. In this report, we validate inhibition of matrigel invasion, ADC, and CAR-T cell applications. Ongoing studies will employ additional antibody engineering for affinity maturation and increased thermostability for superior clinical potential.

## Materials and methods

### Antibody modeling for humanization

Humanization was done by industrial partner Eureka Therapeutics (Emeryville CA) and the nonproprietary aspects are described in patent WO2020227538A1 [39].

### Humanized antibody affinity analysis

Epitope binding assay was performed on ForteBio Octet QK (ForteBio) in 8-channel 96-well plate mode at a shake speed of 1000 rpm. Biotinylated MUC16-peptide at 5 µg/mL was loaded onto the biosensor tips for saturating SA binding sites for 900s. The sensor tips were dipped into kinetics buffer for 300s to remove the non-specific binding and then exposed to the antibodies at 10 µg/mL for association to saturate its binding epitope. Lastly, the sensor tips were moved into kinetics buffer for 1800s to check dissociation.

### Construct design, cloning, and preparation of lentiviral particles

Protein sequence information for the 4H11-scFv were based on descriptions of murine 4H11 from our prior work at Memorial Sloan Kettering Cancer Center (MSKCC) in New York, USA and Eureka Therapeutics (Emeryville CA). The DNA sequences encoding the 4H11 varible regions and a five repeated flexible glycine/serine linker (GGGGS)_5_ between Heavy chain (V_H_: E1-S123) and Light chain (V_L_: D1-R113), [V_H_-linker (GGGGS)_5_-V_L_] were optimized for a scFv mammalian cell expression, and then synthesized commercially (Genewiz). This construct was subcloned into the lentiviral vector with an N-terminal signal peptide. The sequence corresponding to the targeted flexible loop domain (26 residues ^31st^L-G^6th^ from TM) as a MUC16^ecto^ domain was cloned into expression vector pMal-C5X (NEB) for bacterial expression as a Maltose Binding Protein fusion protein. All 4H11-scFv variants and MBP-MUC16 variants were generated by QuikChange site-directed mutagenesis (Stratagene). The 3rd generation lentiviral packaging plasmids; pMDLg/pRRE, pMD2.G, and pRSV-Rev were purchased from Addgene. All lentiviral particles were produced according to the manufacturer’s manual. Briefly, the lentiviral transfer vector and packaging plasmids were co-transfected using LentiTran (Origene) transfection reagent. After 48 h of transfection, the supernatant from the medium was harvested, filtered with 0.45 µM PES filter (ThermoFisher), and the lentiviral particles was stored − 80 ℃ after lentivirus titration, concentration, and stabilization.

### Protein expression and purification

Liter-scale cultures of HEK293T/17 were infected with high-titer viral stocks expressing the 4H11-scFv. The secreted 4H11-scFv from the medium was collected 48–60 h post-infection. The supernatant was dialyzed with Buffer A (50 mM Tris/pH 8.0, 400 mM NaCl) and applied to Ni-NTA agarose beads (nitrilotriacetic acid, Qiagen). After washing with Buffer A supplemented with 20 mM imidazole, bound proteins were eluted with Buffer A supplemented with 500 mM imidazole. The eluted fractions including the scFv proteins were pooled, and the protein was further purified by a Superdex-75 s (GE Healthcare). The 4H11-scFv was concentrated up to ∼ 8 mg/ml using Amicon Ultra centrifugal filters (Millipore) and stored at -80 ℃ until used for further characterization or crystallization. About 1 mg of the purified scFv was labeled with Alexa Fluor 488 carboxylic acid (Life Technologies) according to the manufacturer’s instructions. The fluorescence-labeled 4H11-scFv was further purified by Superdex-75 in 20 mM Hepes buffer with 200 mM NaCl. For production of the variant scFv proteins cloned using the same lentivector, liter-scale suspension cultures of ExpiCHO-S were transfected using the ExpiCHO-S expression system according to the manufacturer’s protocol (Thermo Fisher). The recombinant MBP-MUC16^ecto^ was expressed in the *E. coli* BL21-RIL (DE3) (Novagen) and purified using Amylose resin (NEB) and a Superdex-75 column with an FPLC NGC Quest System (Bio-Rad). The variants of recombinant MBP-MUC16^ecto^ were prepared using a similar protocol.

### Analytical size-exclusion chromatography (SEC) and isothermal titration calorimetry (ITC)

Purified 4H11-scFv and MBP-MUC16^ecto^ were mixed at a molar ratio of ∼ 2.0:1 to assemble the scFv-MUC16^ecto^ complex and incubated at 4 °C for 3 h in buffer containing 50 mM Tris-HCl, pH 8.0, 200 mM NaCl. Each protein of 4H11-scFv and MBP-MUC16^ecto^ was incubated in the same buffer as a control. Protein complex was resolved using Superdex-75 10/300 GL SEC. Excess 4H11-scFv was separated by SEC in the same buffer. ITC was performed at 23 °C on an ITC200 calorimeter from Microcal/GE Life Sciences (Northampton, MA). The scFv samples were used as the titrant in the cell and MUC16 was used as titrants in the syringe. To control for heat or dilution effects, protein samples were dialyzed extensively against the titration buffer (50 mM Tris, pH 8.0, and 400 mM NaCl) prior to each titration. The commercially synthesized MUC16^ecto^ (26 residues) and MBP-MUC16^ecto^ were dissolved in the same buffer. The following concentrations were used for pair-wise titrations: 4H11-scFv (12.8 µM) vs. synthesized MUC16-target (144 µM); 4H11-scFv (12.8 µM) vs. MBP-MUC16^ecto^ (130 µM), respectively. Data were analyzed using the Origin software package provided by the ITC manufacturer. The thermodynamic values reported are the average of three independent experiments.

### Thermal denaturation assay

The thermal stability of 4H11-scFv, 4H11-scFv-MUC16-target peptide were measured using a fluorescence-based thermal shift assay on a Stepone real-time machine (Life Technologies). Immediately before the experiment, the protein (3.2 ug) was mixed with the fluorescent dye SYPRO Orange (Sigma-Aldrich) at multi pH conditions (pH 3.4 through 9.4). The samples were heated from 20 to 95 °C in ∼ 50 min. The midpoint of the protein-melting curve (*T*_*m*_) was determined using the analysis software provided by the instrument manufacturer. The data obtained from three independent experiments were averaged to generate the bar graph. The *T*_*m*_ of 4H11-scFv-MUC16^ecto^ at pH 3.4 could not be determined due to high fluorescence signal at starting temperature. The *T*_*m*_ of MUC16^ecto^ and the mutants were measured using a similar protocol.

### Crystallization

Initial crystallization screens were performed using a Phoenix crystallization robot (Art Robbins Instruments) and high-throughput crystallization screen kits (Hampton Research, Qiagen, or Emerald BioSystems), followed by extensive manual optimization. The best single crystals were grown at 18 °C by the hanging-drop vapor-diffusion method in a 1:1 (v/v) ratio of protein and reservoir, as follows. (1) 4H11-scFv was crystallized with a reservoir solution composed of 0.1 M sodium citrate tribasic dihydrate (pH 5.0) and 20% polyethylene glycol (PEG) 4 K. Micro-seeding was necessary to obtain single crystals. (2) 4H11-scFv-MUC16-target complex was crystallized using a reservoir of 0.1 M sodium citrate tribasic dihydrate (pH 5.0), 10 mM barium chloride dihydrate, and 27% methoxypolyethylene glycol 5000 (PEG MME 5 K).

### Diffraction data collection and structure determination

The crystals were cryo-protected in the original mother liquor supplemented with 20% (v/v) glycerol and flash-frozen in liquid nitrogen. X-ray diffraction data was collected at NE-CAT beamline 24-ID-E using an Dectris EIGER 16 M detector at a wavelength of 0.979180 Å. Data was automatically indexed and reduced using XDS and Aimless as implemented in RAPD (https://github.com/RAPD/RAPD), the data-processing pipeline implemented at NE-CAT. Data collection statistics are summarized in Supplementary Table 1. The structure of the 4H11-scFv antibody was determined by Molecular Replacement (MR) software Phaser using V_H_ (PDB: 6ATT) and V_L_ (PDB: 3OKK) as the search models. Subsequent structure of the 4H11-scFv in complex with MBP-MUC16^ecto^ was determined by molecular replacement using the determined 4H11-scFv structure as a model, and an MBP (PDB: 3VD8) as the search model. An MBP-MUC16^ecto^ was modeled into the corresponding structure during the refinement based on the 2Fo-Fc electron density maps and a combination with a partial peptide structure of the SEA domain (PDB: 1IVZ). The manual model building and refinements were performed in COOT and PHENIX in an iterative manner until satisfactory model statistics was achieved. The refinement progress was monitored with the free R value using a 5% randomly selected test set. The structures were validated through MolProbity and showed excellent stereochemistry. Structural refinement statistics are listed in Supplementary Table S1. PDB accession codes for newly reported structures are PDB ID 8VRS and PDB ID 8VRR.

### Pull-down assay

A series of MBP pull-down assays were performed in vitro to determine a physical interaction among MBP-tagged MUC16-target (wt), its alanine mutants (D25A/R24A, D18A/G17A, or D25A/R24A/D18A/G17A) as a bait, 4H11-scFv (wt) and the mutants (S53A/D106A, Y246A/N247A, or S53A/D106A/Y246A/N247A) as a prey were performed in parallel in a buffer containing 50 mM Tris-HCl, pH 8.0, 400 mM NaCl, 1 mM EDTA and 1 mM DTT using Amylose resin, which is an affinity matrix used for the isolation of proteins fused to MBP at 4 °C for 3 h. The resins were washed three times before boiling with the addition of SDS sample buffer, and further analyzed by 4–20% gradient SDS-PAGE. Each pull-down was performed in triplicate and a representative SDS-PAGE gel is shown. MBP-tagged Protein baits were pre-incubated with Amylose resins at 4 °C for 2 h, and unbound proteins was washed away. The resins were equally divided for repeated experiments into small aliquots where each has ∼ 20 µg of bound protein bait. 4H11-scFv (wt) or the mutated 4H11-scFv prey proteins used at 2-fold molar excess over MBP- MUC16^ecto^ were added. All pull-down assays were performed using the same protocol.

### Statistical analysis

Survival curves were analyzed using Mantel–Cox (log-rank) test and other analysis were performed using unpaired two-tailed t test (*p* value < 0.05 considered as significant). All calculations were performed using Prism 7 (GraphPad) software. Data represent means ± SEM.

### Electronic supplementary material

Below is the link to the electronic supplementary material.

Supplementary Material 1

## Data Availability

All data are available in the main text or the supplementary materials. PDB accession codes for newly reported structures are PDB ID 8VRS and PDB ID 8VRR.
