# Supplementary material for: Structural basis for antibody recognition of the proximal MUC16 ectodomain
Source: J Ovarian Res. 2024 Feb 19;17:41. doi: 10.1186/s13048-024-01373-9 (PMC10875768; doi:10.1186/s13048-024-01373-9)
Supplement: Supplementary file 1 — Supplementary Material 1 [file 13048_2024_1373_MOESM1_ESM.pdf]

Supplementary Materials for

Structural basis for antibody recognition of the proximal MUC16 ectodomain

Lee *et al.*

*Corresponding author. Email: oyeku@mgh.harvard.edu

This PDF file includes:

Supplementary Methods
Figs. S1 to S4
Tables S1 to S4

Supplementary Methods

Cell lines

HEK293T/17 and ExpiCHO-S (Thermo Fisher Scientific) cell lines were used for protein expression. Ovarian cancer cell lines SKOV3 (MUC16 negative control), SKOV3-MUC16^{ecto} (isogenic MUC16 positive cell line), OVCAR3 (MUC16 positive), OVCAR3$^{MUC16k/o}$ (MUC16 negative), OVCA-433 (MUC16 positive), and CAOV3 (MUC16 positive) were used for *in vitro* and *in vivo* experiments (*24, 25, 27*). SKBR3 (breast cancer) cell line was purchased from ATCC. All cell lines were maintained in their original culturing conditions according to supplier guidelines and tested negative for mycoplasma contamination. Stable HEK293T/17 cells were generated by lentiviral transduction with a construct expressing pLenti-C-Myc-DDK-IRES-Puro (Origene), followed by selection with puromycin. All cell lines have been verified and are routinely tested for mycoplasma.

Functional analysis of humanized antibodies

Matrigel invasion assay. Antibody inhibition of basement membrane invasion was determined via Matrigel invasion assay, as previously described (*24, 25*). Briefly, OVCAR3, OVCAR-433 and CAOV3 cells were pretreated with a 10 µg/ml of the indicated antibodies prior to exposure to the Matrigel invasion chambers. Control chambers were set up with cells that had not been exposed to antibody. Each insert contained 10,000 cells per chamber. Both experimental and control chambers were set up in three biologic replicates. The number of invading cells was counted after a 48-hr. incubation period.

Antibody Drug Conjugate Assay. SKOV3, SKOV3-MUC16^{ecto} OVCAR3 and OVCAR3$^{MUC16k/o}$ tumor cells were incubated with various antibody targeting preparations using Oyo-link (Alphathera, Philadelphia PA) using the manufacturer supplied protocol (1). Cytotoxicity was subsequently evaluated via luciferase-based cytotoxicity assays as above ≥ 3 times.

Chimeric Antigen Receptor T-cell assay. Human T cells were derived from fresh blood-derived leukocyte concentrate (Leukopack) obtained from Research Blood Components LLC (Waltham, MA). Mononuclear cells were separated using density gradient centrifugation with Accuprep (axis-Shield PoC AS, Oslo, Norway). T cells were isolated, activated, and expanded with 2µl/mL PHA (Sigma Aldrich, St. Louis, MO). T cells were cultured in RPMI 1640 in the presence of 10 ng/mL recombinant human IL-2 (Proleukin). Viable cells were enumerated using flow cytometry and CAR T-cell transduction efficiency was determined using anti-F(ab)2 antibody (R&D Biosystem, F0101B) following manufacturer's protocol. For 4-hr cytotoxicity experiments, 4H28ζ CAR T-cells or controls were cultured with indicated tumor cells and evaluated for cytotoxicity by standard ^{51}Cr release assay as previously described (*29*). For 72-hr luciferase-based cytotoxicity assays, 4H28ζ CAR T-cells or controls were cultured with indicated T-cells and subsequently mixed with luciferase assay reagent (Promega). Luminescence of the lysates was analyzed using a plate spectrophotometer. Specific cytolysis was calculated using the formula; % specific lysis = 100 × (sample lysis –spontaneous lysis)/(maximal lysis –spontaneous lysis). To evaluate cytokine secretion from cocultured cells, indicated CAR T-cells cocultured with SKOV3-MUC16^{ecto} or OVCAR3 cells, untransduced T-cells, and tumor cells alone were cultured for 72 hrs. Supernatant was collected and centrifuged at 1800 rpm for 10-15 mins to remove any contaminating cells or debris. The cell-free supernatant was then transferred to a fresh tube and frozen at -80°C until further analysis. Cytokine detection was performed using the

High Sensitivity 9-Plex Human ProcartaPlex™ Panel (ThermoFisher Scientific, EPXS090-12199-901), and the Luminex 200 system according to the manufacturers protocol. For *in vivo* studies, female NSG mice age 8-12 weeks were purchased from the Massachusetts General Hospital COX7 animal facility. $1x10^7$ SKOV3-MUC16^{ecto} tumor cells were injected intraperitoneally (i.p.) on D0, and animals were untreated, or treated with 2 $x10^6$ CAR T-cells i.p. on day 14. All mice were monitored for survival and were euthanized when showing signs of distress. All murine studies were done in the context of a Massachusetts General Hospital Institutional Animal Care and Use Committee approved protocol (2018N000207).

Cell-based assay for internalization of 4H11-scFv into the MUC16-overexpressed cells
The purified 4H11-scFv was conjugated to Alexa fluor® 488 fluorescent dye (Alexa-4H11-scFv) using a labeling kit in accordance with the manufacturer's protocol (Invitrogen). Two MUC16 negative control (HEK293T and SKOV3) and two MUC16-positive (OVCAR3 and SKBR3) cell lines were seeded in a 6-well plate and incubated with fresh culture medium for 24 hrs. Each cell was fixed, permeabilized, and blocked for 1 hr using ImageiT Fixation kit (Life Technologies) as per manufacturer's instruction and then, incubated with Alexa-4H11-scFv (10 nM) for 30 min at 25 °C and washed three times with PBS buffer. Images were collected using a confocal microscope (Nikon Eclipse TE2000-S microscope). To investigate the internalization of 4H11-scFv antibody, OVCAR3 and SKBR3 were plated on independent 6-well plates at a density of 2 x 10^5 and left 24 hrs. The medium was removed, washed twice with PBS buffer and replaced with Live Cell Imaging Solution (Invitrogen). The Alexa-4H11-scFv was first added for 30 min to the each well and washed twice with the imaging solution. The Alexa-350-WGA (5 ug/mL), binding to sialic acid and N-acetylglucosaminyl residues of cell membranes, was then prepared by manufacturer's protocol. The imaging solution with Alexa-350-WGA was removed after 20 min incubation and then washed three times. Fluorescent images for scFv tracking were taken every 12-hr, from 2hr - 48 hr, using the same confocal microscope.

Reference:

1. Utilizing Second-Generation Antibody-Drug Conjugates for the Management of Metastatic Breast Cancer: A Comprehensive Review of Glembatumumab Vedotin (CDX-011, CR011-vcMMAE)." C. Vaklavas et al., BioDrugs, June 2014, V28, Issue 3, P253-263.

Supplementary Results:

Figure S1

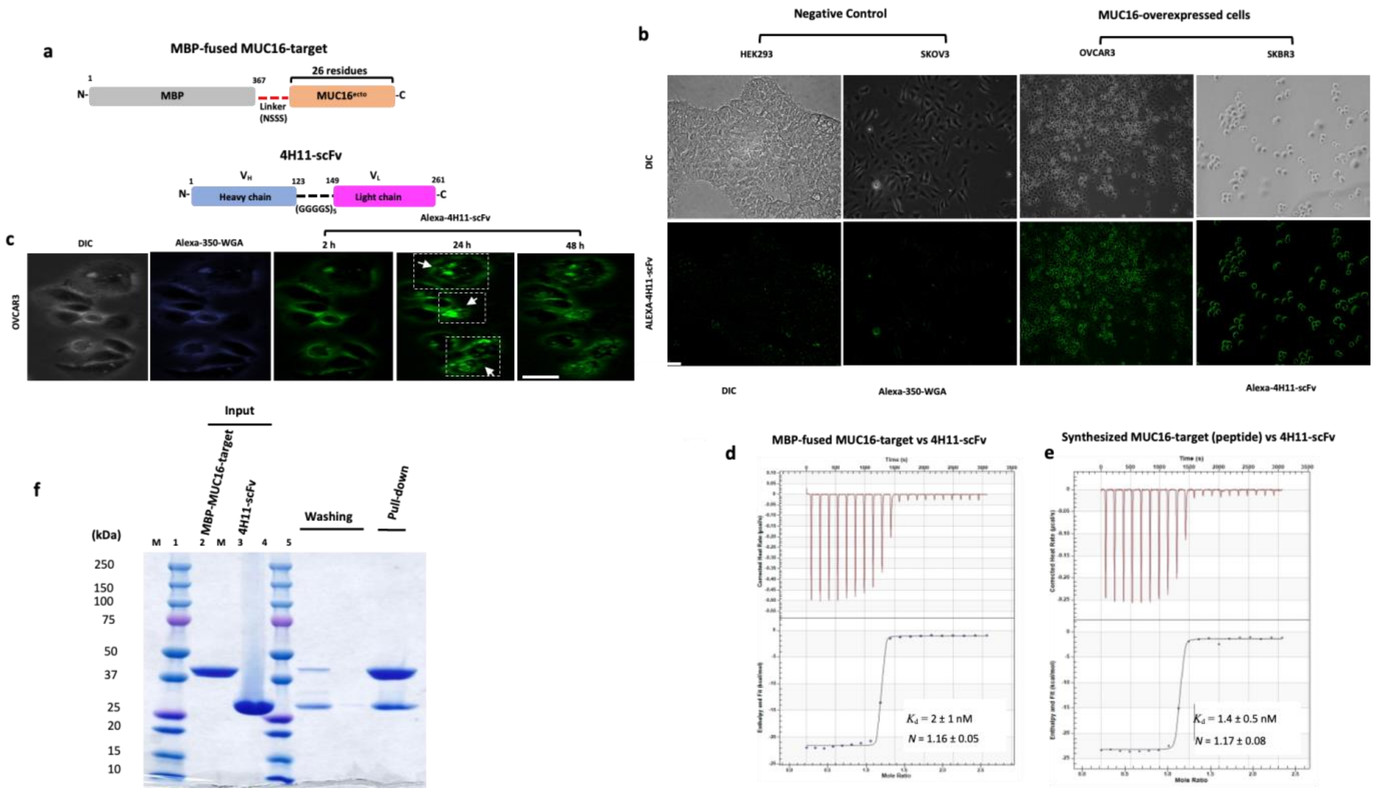

Figure S1. Behavior and binding of MBP-fused MUC16-target with 4H11-scFv. (a) A schematic diagram showing the domain organizations of MBP-fused MUC16-target and the 4H11-scFv. MBP, maltose binding domain (grey); linker composed of NSSS (red dots); MUC16-target composed of 26 residues (orange); Heavy chain of 4H11-scFv (skyblue); linker composed of (GGGGS)5 repeats (black dots); Light chain of 4H11-scFv (pink). **(b)** MUC16 overexpressed in OVCAR-3 and SKBR3 cancer cell lines can bind to 4H11-scFv that was conjugated to Alexa fluor® 488 fluorescent dye (Alexa-4H11-scFv) while negative control cell lines, HEK293 and SKOV3, showed no binding. The fixed cell imaging; DIC images (upper panel) and immunofluorescence images of 4H11-scFv (lower panel). **(c)** Live cell imaging of OVCAR3 (10 nM, 12 hrs). DIC image (left panel), Alexa-350-WGA that binds to sialic acid and Nacetylglucosaminyl residues of cell membranes and Alexa-4H11-scFv (right panel), the white arrows in the white dot boxes indicate the 4H11-scFv vesicles internalized into the cell. Scale bar: 30 µm. **(d,e)** Thermodynamic studies of isothermal titration calorimetry (ITC). Representative titration profiles are shown for the binding of 4H11-scFv to the MBP-fused MUC16-target (Kd = 2 ± 1 nM; DH = -19.9 ± 0.6 kcal/mol; -TDS = 7.9 ± 0.8 kcal/mol; N = 1.16 ±0.05) or the synthesized MUC16-target peptide (Kd = 1.4 ± 0.5 nM; DH = -21.2 ± 0.5 kcal/mol; - TDS = 9.1 ± 0.6 kcal/mol; N = 1.17 ± 0.08). The thermodynamic values reported are the average of three independent experiments (mean ± SD). **(f)** MBP pull-down between MBP-

tagged human MUC16-target and the antibody, 4H11-scFv; MBP-MUC16-target (lane 1), 4H11-scFv (lane 2) as input, washing steps (lane 3, lane 4), and output of pull-down (lane 5). Input molar ratio was 1:3 (MBP-MUC16 : 4H11-scFv) and unbound proteins were eliminated at twice washing steps.

Figure S2

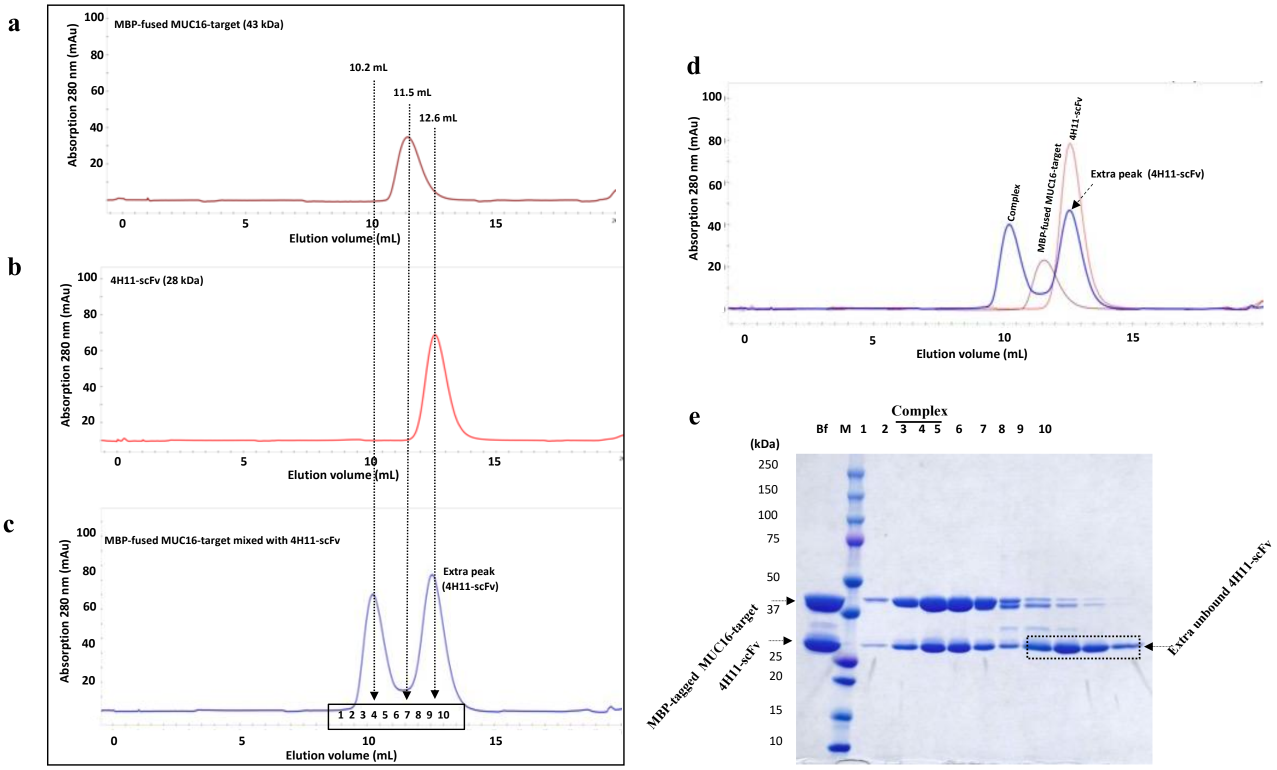

Figure. S2. Characterization of interactions between 4H11-scFv and MUC16-target by size-exclusion chromatography (SEC, Superdex 75 column). The separately expressed and purified the MBP-tagged MUC16-target **(a)** and 4H11-scFv **(b)** formed a stable complex **(c)** when they are mixed, MBP-tagged MUC16-target (15 uM) and 4H11-scFv (30 uM) were mixed at 4 °C during 3 hrs. The eluted peak positions a, b, and c are 11.5 mL (brown), red (12.6 mL), and blue colors (10. 2mL), respectively. **(d)** A superposition of elution profiles, the extra 4H11-scFv (blue) peak shown at the same position with the 4H11-scFv eluted. (e) The peak SEC fractions (# 1-10) were analyzed by SDS-PAGE and Coomassie staining. The fraction numbers are labeled on the top. “Bf” and “S” stand for the before injection and protein marker being analyzed, respectively. In panel e, the lower band 4H11-scFv (28 kDa) can bind to the MBP-tagged MUC16-target (43 kDa) composed of 26-residues including a flexible loop with two β-turns and one β-hairpin structures, that can bind to the CDR2 and 3 of the heavy chain (V_H) and CDR1 and 3 of the light chain (V_L) of 4H11-scFv. The complex fractions are shown around 10.2 mL peak (lane 3 – 5) and excess 4H11-scFv protein (black dot) was eluted around 12.6 mL position (lane 7 – 10).

Figure S3

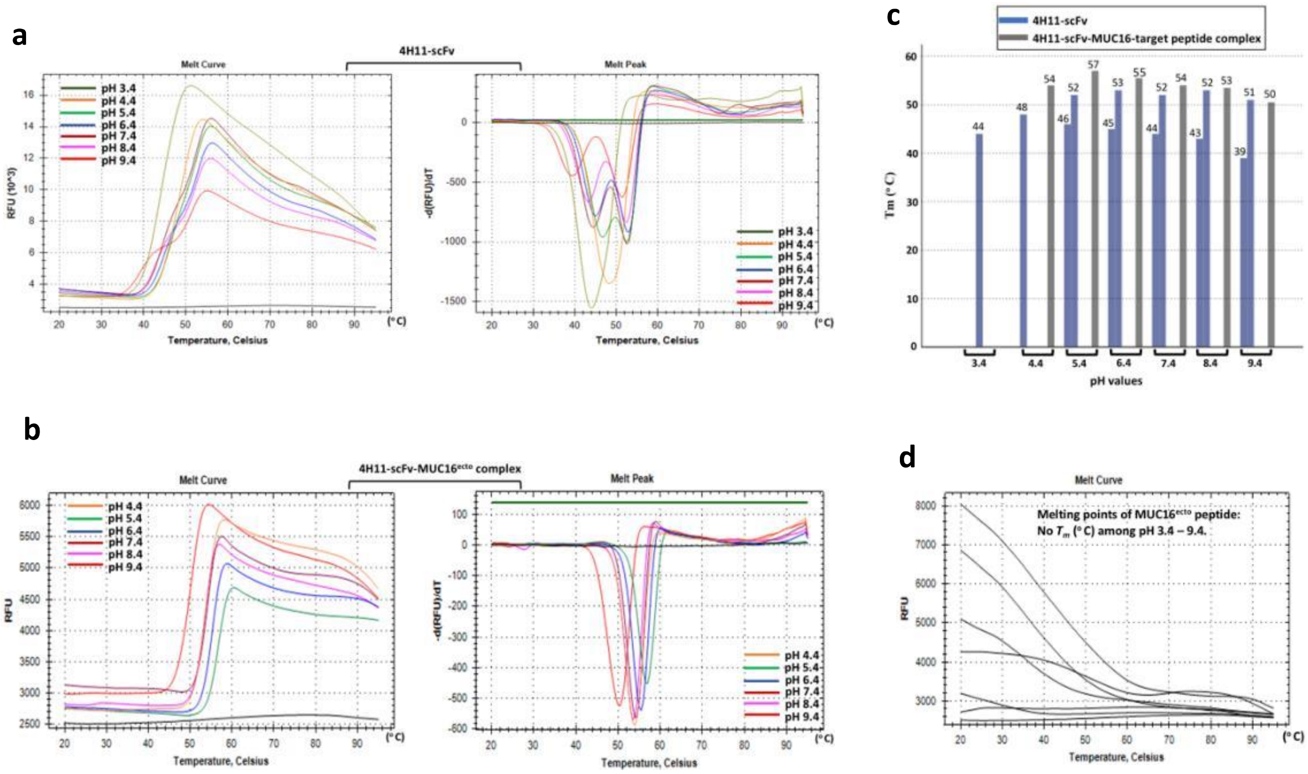

Figure. S3. Thermal stability of 4H11-scFv or 4H11-scFv-MUC16 complex at acidic, neutral and basic pH. (a, b) The thermal stability of wild type 4H11-scFv or 4H11-scFv-MUC16 was measured using a fluorescence-based thermal shift assay on a CFX96 real-time system (Bio-Rad). Protein melting was monitored using a hydrophobic dye, SYPRO Orange (Sigma-Aldrich), as the temperature was increased in a linear ramp form 20 ^{o}C to 95 ^{o}C. The midpoint of the protein-melting curve (T_m) was determined using the software provided by the instrument manufacturer. The data are presented as mean ± S.D., n = 3. (a) The T_m of WT 4H11-scFv at pH 3.4 and 4.4 showed only one melting point while the T_m among pH 5.4 – 9.4 showed two melting points, implying either V_H or V_L may show a higher stability. However, the 4H11-scFv in complex with MUC16-target peptide (26 residues) showed the higher T_m by 1 ^{o}C to 6 ^{o}C except for pH 9.4, it showed 1 ^{o}C lower T_m than WT protein. The T_m of the complex at pH 3.4 could not be determined due to significant protein unfolding at the starting temperature. **(c)** The 2-D bar chart represents the T_m comparison of "a" and "b" according to pH values. 4H11-scFv is blue color and 4H11-scFv-MUC16-target complex is grey color, respectively. **(d)** The T_m measurement of MUC16-target peptide itself among multiple pH values. The peptide itself did not have any effect on the T_m of the complex.

Figure S4

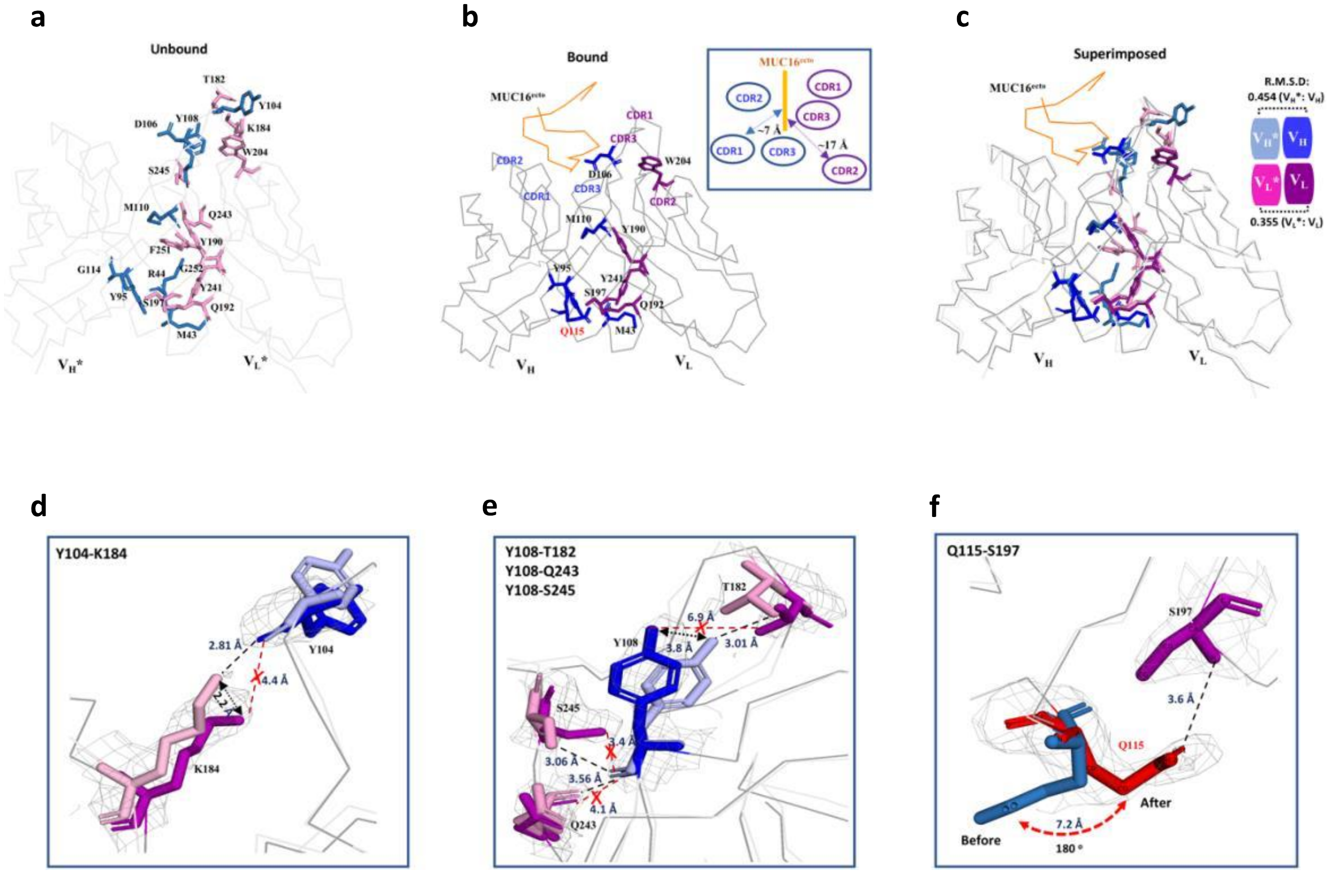

Figure. S4. The conformational dynamics of antibody V_H-V_L binding interface residues depending upon antigen binding. **(a)** 4H11-scFv itself can be extensively stabilized by V_H (skyblue) and V_L (pink) interaction. There are 11 hydrogen bonds based on our X-ray structure between V_H and V_L. **(b)** The interface residues showed dramatic conformational changes depending upon MUC16 binding (orange), showing the reduced 6 hydrogen bonds with different atom-to-atom distances and a new hydrogen bond between Q196 (red) of V_H and S197 of V_L compared to antibody itself. **(c)** The superimposed line model between bound and unbound status. Root-mean-square-deviation (R.M.S.D) between the backbone atoms of superimposed proteins was 0.454 (V_H*: V_H) or 0.355 (V_L*: V_L), respectively. (d-f) The representative residues with 2Fo-Fc electron density map (contoured at 1.0 σ) that showed the conformational dynamics. (d) The hydrogen bond between Y104 (V_H) and K184 (V_L) was broken (red “x”) depending upon MUC16 binding due to the movement of K184 by 2.2 Å (black bi-arrow) with the orientation change of Y104 aromatic ring. The measured distance between the moved K184 and Y104 was 4.4 Å with no contact. **(e)** Y108 (V_H) was moved toward MUC16 by 3.3 Å and the previous hydrogen bonds formed with V_L residues (T182, Q243, and S245) were broken (red ”x”) and it formed new hydrogen bonds with water-mediated interactions (see fig 5g and table S-2 for detailed version). **(f)** A new hydrogen bond was formed

between Q115 (V_H) and S197 (V_L). The MUC16 binding triggers a ~180° rotation of the sidechain (red) to form a hydrogen bond with S197.

Table S1: Data collection and refinement statistics

	4H11-scFv-MUC16^{ecto} complex	4H11-scFv
**Data collection**		
Space group	P1 21 1	I 21 21 21
Cell dimensions		
a, b, c (Å)	59.81, 112.634, 110.81	51.592 112.795 150.668
α, β, γ (°)	90, 101.253, 90	90 90 90
Resolution (Å)	78.21-2.46 (2.55-2.46)[a]	75.33 - 2.36 (2.445 - 2.36)[a]
R_{merge}	0.1064 (0.8136)	0.09106 (1.216)
CC1/2	0.994 (0.676)	0.998 (0.713)
I/σ(I)	9.35 (1.46)	15.48 (1.54)
Completeness (%)	99.08 (99.20)	99.4 (99.9)
Redundancy	3.5 (3.5)	3.4 (3.3)
**Refinement**		
Resolution (Å)	56.43-2.47	75.33 - 2.36
Total no. of reflections	180138	16317
Reflections used for Refinement	51240	1636
R_{work}/R_{free}	0.186/0.242	0.202/0.247
No. atoms	10114	1904
macromolecules	9688	1817
Ligand/ion	-	-
Water	148	87
Protein residues	1252	236
Overall B-factors	42.1	43.3
R.m.s. deviations		
Bond lengths (Å)	0.008	0.007
Bond angles (°)	0.961	0.952
Ramachandran favored (%)	96.29	94.83
Ramachandran allowed (%)	3.15	4.74
Ramachandran outliers (%)	0.56	0.4

Values in parentheses are for the highest-resolution shell.
Each dataset was derived from a single crystal.

Table S2: Dynamic rearrangement of V_H-V_L interface before/after binding to MUC16^{ecto}

Heavy chain-Light chain interaction before binding to MUC16-target

Heavy chain residues	Light chain residues	Type of interaction	Distance (Å)
**MET 43 [O]**	**TYR 241 [OH]**	Hydrogen bond	3.53
ARG 44 [NH1]	GLY 252 [O]	Hydrogen bond	3.22
ARG 44 [NH2]	PHE 251 [O]	Hydrogen bond	3.13
ARG 44 (side chain)	PHE 251 (aromatic)	cation- π interaction	5.6
**TYR 95 [OH]**	**GLN 192 [OE1]**	Hydrogen bond	2.97
TYR 104 [O]	LYS 184 [NZ]	Hydrogen bond	2.81
**ASP 106 [O]**	**TRP 204 [NE1]**	Hydrogen bond	2.92
TYR 108 [O]	GLN 243 [NE2]	Hydrogen bond	3.56
TYR 108 [O]	SER 245 [OG]	Hydrogen bond	3.06
TYR 108 [OH]	THR 182 [OG1]	Hydrogen bond	3.01
**MET 110 [N]**	**TYR 190 [OH]**	**Hydrogen bond**	**2.95**
**GLY 114 [O]**	**SER 197 [OG]**	**Hydrogen bond**	**2.31**

After complex ➔

Heavy chain-Light chain interaction after binding to MUC16-target

Heavy chain residues	Light chain residues	Type of interaction	Distance (Å)
MET 43[O]	TYR 241[OH]	Hydrogen bond	2.92
TYR 95[OH]	GLN 192[NE2]	Hydrogen bond	3.04
ASP 106[O]	TRP 204[NE1]	Hydrogen bond	3.03
MET 110[N]	TYR 190[OH]	Hydrogen bond	2.67
GLY 114[O]	SER 197[OG]	Hydrogen bond	2.39
GLN 115[OE1]	SER 197[N]	Hydrogen bond	3.63

Table S3: Analyzing of torsional angles from L31st to P14th of MUC16^{ecto}

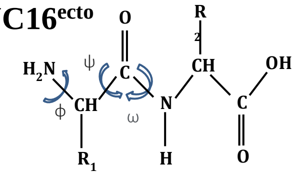

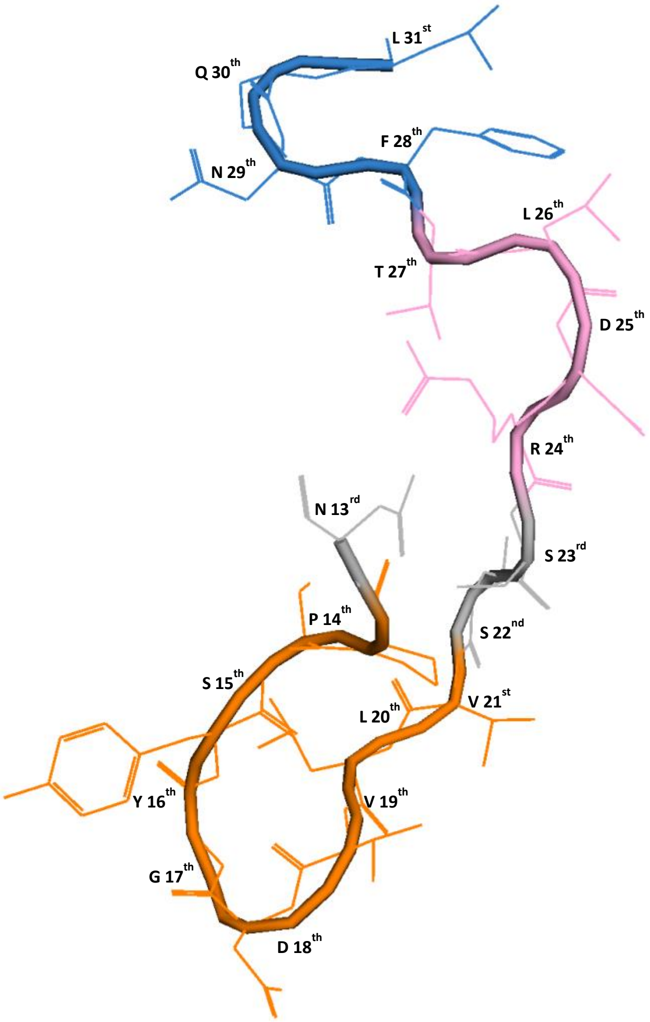

	Phi (φ)	Psi (ψ)	Omega (ω)
Lys 31	None	None	15.7
Gln 30	-57.4	-43.0	-2.8
Asn 29	-130.3	54.5	-8.0
Phe 28	-57.4	137.8	13.9
Thr 27	-116.1	125.0	0.9
Leu 26	-57.8	-65.1	5.1
Asp 25	-110.3	95.5	3.0
Arg 24	-89.8	116.5	4.2
Ser 23	-81.1	-35.4	4.1
Ser 22	54.3	32.0	0.8
Val 21	-133.6	147.3	-13.0
Leu 20	-116.4	141.9	5.5
Val 19	-117.0	112.8	1.7
Asp 18	51.7	38.2	-2.6
Gly 17	84.7	12.9	2.1
Tyr 16	-139.9	145.2	-8.3
Ser 15	-64.1	144.6	-14.3
Pro 14	-70.7	135.2	15.7
Asn 13	None	None	None

β turn (Lys 31 – Asn 29)

β turn (Thr 27 – Arg 24)

β hairpin (Leu 20 – Pro 14)

Table S4: Protein-protein interaction in the 4H11-scFv-MUC16-target complex

V_H or V_L	CDR position	4H11-scFv residues	MUC16-target residues	Type of interaction	Distance (Å)	
V_H	CDR 2	SER 52[OG]	ASP 25th [OD1]	Hydrogen bond	2.47	
V_H	CDR 2	SER 53[N]<br>SER 53[N]<br>SER 53[OG]<br>SER 53[OG]	ARG 24th [O]<br>ASP 25th [OD1]<br>ARG 24th[N]<br>LEU 26th[O]	Hydrogen bond<br>Hydrogen bond<br>Hydrogen bond<br>Hydrogen bond	2.98<br>3.67<br>2.88<br>3.17	
V_H	CDR 2	ALA 54[N]	ASP 25th[OD1]	Hydrogen bond	3.03	
V_H	CDR 3	ASN 103 [OD1]	GLN 30th [NE2]	Hydrogen bond	2.70	
V_H	CDR 3	ASP 106[OD2]<br>ASP 106[OD2]<br>ASP 106[OD2]	SER 22nd[OG]<br>ARG 24th[NH1]<br>ARG 24th [NH1]	Hydrogen bond<br>Hydrogen bond<br>Salt-bridge	2.71<br>3.20<br>3.49	
		ASP 106[OD2]	SER 22nd [N]	Water (W 2)-mediated interaction	OD2-W 2: 3.6	W 2- N: 2.9
V_H	CDR 2	TYR 108[OH]	SER 15th[OG]	Water (W 1)-mediated interaction	OH-W 1: 3.2	W 1- OG: 2.3
			SER 23rd[OG]	Water (W 1)-mediated interaction	OH-W 1: 3.2	W 1- OG: 2.7
V_L	CDR1	SER 180[OG]	TYR 16th [OH]	Hydrogen bond	3.23	
		SER 180 [OG]	GLY 17th [O]	Water (W 3)-mediated interaction	OG-W 3: 2.9	W 3- O: 2.8
		SER 180 [N]	GLY 17th [O]	Water (W 3)-mediated interaction	N-W 3: 2.7	W 3- O: 2.8
V_L	CDR3	TYR 246[O]<br>TYR 246[O]<br>TYR 246[OH]	GLY 17th [N]<br>ASP 18th [N]<br>ASP 18th [O]	Hydrogen bond<br>Hydrogen bond<br>Hydrogen bond	2.87<br>3.80<br>3.56	
V_L	CDR3	ASN 247[OD1]	ASP 18th [N]	Hydrogen bond	2.88	
V_L	CDR3	LEU 248[N]	LEU 20th [O]	Hydrogen bond	2.75	
